# Proteomic Signatures of Noise‐Induced Hearing Loss in the Mouse Cochlea

**DOI:** 10.1002/cbin.70218

**Published:** 2026-10-01

**Authors:** Ana Carla Batissoco, Danillo Alencar‐Coutinho, Ana Carolina Ferreira‐Scatone, Stella Diogo‐Cavassana, Jeanne Oiticica, Karina Lezirovitz, Ricardo Ferreira Bento

**Affiliations:** ^1^ Laboratório de Otorrinolaringologia/LIM 32, Hospital das Clínicas HCFMUSP, Faculdade de Medicina Universidade de São Paulo São Paulo São Paulo Brazil; ^2^ ENT Department Hospital das Clínicas da Faculdade de Medicina da Universidade de São Paulo São Paulo São Paulo Brazil

**Keywords:** auditory synaptopathy, biomarkers, cochlear proteomics, noise‐induced hearing loss, precision audiology, translational targets

## Abstract

Hearing loss affects over 1.5 billion people worldwide and has substantial social, educational, and economic consequences. Although genetic studies have identified numerous hearing‐loss‐associated genes, the molecular changes accompanying noise‐induced hearing loss (NIHL) remain incompletely understood. We characterized the adult mouse cochlear proteome following damaging noise exposure using nano‐LC–MS/MS and an integrative bioinformatics workflow. Comparative profiling of normal‐hearing and NIHL cochleae identified 1742 proteins and an 80‐protein NIHL‐associated signature comprising 49 quantitatively defined differentially abundant proteins and 31 condition‐specific proteins. These proteins were organized into five functional groups encompassing metabolic and mitochondrial function, protein synthesis and ribonucleoprotein complexes, structural and synaptic organization, cell polarity and cytoskeletal remodeling, and regulatory and stress‐responsive functions. Representative alterations included reduced HSPA9, a mitochondrial chaperone involved in protein homeostasis; changes in the RNA‐associated proteins PRMT1 and CIRBP; reduced HAPLN1, associated with extracellular matrix organization; and altered MAP2K2, a signaling protein related to cell polarity; and increased SYNJ2. Comparison with an independent cochlear proteomic data set identified 39 of the 80 proteins as dysregulated, with 22 showing concordant directional changes. Single‐cell transcriptomic mapping provided cellular context for 74 of the 80 protein‐associated genes, while human genetic and disease annotations supported the prioritization of selected proteins associated with hearing or ear phenotypes. Together, these findings define a proteomic landscape of the noise‐exposed cochlea and identify molecular candidates and cellular processes for further investigation of NIHL.

**Abbreviations**
ABR: auditory brainstem responses
CCs: circulating cells
DAPs: differentially abundant proteins
DFN: deafness loci
FC: fold change
FDR: false discovery rate
GO: Gene Ontology
HCs: hair cells
HI: hearing impairment
HL: hearing loss
HPO: Human Phenotype Ontology
LFQ: label‑free quantification
LW: lateral wall
MONDO: Mondo Disease Ontology
MPO: Mammalian Phenotype Ontology
MS: mass spectrometry
Nano‑LC–MS/MS: nano‑liquid chromatography tandem mass spectrometry
NH: normal hearing
NIHL: noise‐induced hearing loss
OMIM: Online Mendelian Inheritance in Man
ORPHA: ORPHANET Rare Disease Ontology
PPI: protein–protein interaction
scRNA‑seq: single‑cell RNA sequencing
SCs: supporting cells
SGN: spiral ganglion neurons
Tbio: biologically characterized target
TCA: tricarboxylic acid
Tchem: target with known bioactive chemistry
Tclin: clinically validated target
Tdark: poorly characterized target

## 1 Introduction

A fully functional auditory system is fundamental for effective communication and accurate environmental perception. Impaired sound‐to‐neural‐signal transduction leads to hearing loss (HL), imposing substantial social, educational, vocational, and economic burdens, even when clinically mild (Stelma and Bhutta 2014). HL is one of the most common sensory disorders worldwide and shows remarkable clinical and etiological heterogeneity (Stelma and Bhutta 2014). Genetic factors are estimated to account for approximately 50%–60% of HL cases, with nearly 70 percent classified as nonsyndromic (Sheffield and Smith 2019). However, despite the mapping of more than 200 genomic loci, the number of identified causative genes remains limited to approximately 170 (Walls et al. 2025). This discrepancy indicates that critical gene products and molecular pathways involved in auditory function remain unidentified, highlighting the need for integrative approaches that extend beyond genomics to uncover novel mechanisms and therapeutic targets for HL. In addition to genetic contributions, environmental and acquired factors such as noise exposure, infections, and ototoxic medications, together with systemic conditions such as diabetes and age‐related degeneration, further contribute to the multifactorial etiology of HL (Bowl and Dawson 2019; Xu et al. 2024; Prasad et al. 2024).

Globally, an estimated 1.57 billion people, approximately one in five worldwide, had HL in 2019, and this number is projected to increase to 2.45 billion by 2050, largely driven by population aging (Haile et al. 2021). In Latin America alone, over 62 million people live with moderate to profound HL, with adult prevalence in countries such as Brazil and Mexico reaching up to 26%. This high burden is largely driven by preventable environmental causes and persistent inequalities in access to hearing health care services (Lezirovitz and Mingroni Netto 2022).

Beyond its sensory consequences, untreated HL has been associated with an increased risk of cognitive decline and neurodegenerative diseases. Meta‐analyses have demonstrated a 59% increased risk of dementia and an almost twofold increase in the incidence of Alzheimer's disease among individuals with HL (Ford et al. 2018; Jafari et al. 2019). Proposed mechanisms include increased cognitive load, reduced social engagement, and neuroplastic alterations resulting from prolonged sensory deprivation. Importantly, early hearing rehabilitation using hearing aids or cochlear implants has been associated with a 19% reduction in the hazard of long‐term cognitive decline (Yeo et al. 2023).

The cochlea plays a central role in auditory signal transduction and is particularly vulnerable to structural and functional damage (Fettiplace 2017; Fetoni et al. 2019). Sensorineural HL, the most prevalent form, commonly involves the degeneration of cochlear hair cells or their associated neurons (Fettiplace 2017). Although transcriptomic studies have provided valuable insights into cochlear gene expression, mRNA expression levels do not consistently reflect protein abundance or functional activity due to extensive post‐transcriptional and post‐translational regulatory mechanisms (Greenbaum et al. 2003). In contrast, proteomic approaches, particularly those based on mass spectrometry (MS), enable direct investigation of protein networks and biological pathways critical for cochlear integrity and auditory function (Darville and Sokolowski 2013; Hickox et al. 2014; Lamas and Malgrange 2016).

Noise‐induced hearing loss (NIHL) represents the second most common cause of environmentally induced HL worldwide, posing a major public health concern, particularly among young adults and occupational groups exposed to high‐intensity noise. Globally, approximately 1.1 billion individuals are at risk (Ding et al. 2019; Miao et al. 2022). Although outer hair cells are the primary targets of noise‐induced damage, inner hair cells, synaptic connections, and cochlear neurons are also susceptible. Noise exposure triggers cochlear inflammation, oxidative stress, metabolic dysregulation, and synaptopathy, culminating in progressive and often irreversible auditory dysfunction (Fetoni et al. 2019; Ding et al. 2019).

Despite the clinical and public health relevance of NIHL, the cochlear proteome under pathological noise exposure remains insufficiently characterized. This gap is largely attributable to technical challenges, including the small size of the cochlea, marked cellular heterogeneity, and limited availability of biological material, which have historically constrained comprehensive proteomic analyses (Noble et al. 2018; Khorrami et al. 2025). Recent advances in mass spectrometry‐based proteomics, together with the increasing availability of complementary transcriptomic and proteomic datasets, have expanded the capacity to characterize molecular alterations and dysregulated biological pathways associated with NIHL (Jongkamonwiwat et al. 2020; Miao et al. 2022; Qiu et al. 2023). Nevertheless, the molecular alterations underlying noise‐induced cochlear dysfunction remain incompletely understood, particularly at the level of protein abundance and their integration with cellular and functional context.

In this study, we aimed to characterize proteomic alterations in cochleae from adult mice with normal hearing (NH) and NIHL using high‐resolution mass spectrometry (MS) combined with integrative bioinformatic analyses. By identifying protein signatures and biological processes associated with noise‐related cochlear damage, this work aims to provide a basis for further investigation of the molecular processes involved in NIHL.

## 2 Methodology

### 2.1 Animals

All experimental procedures were approved by the Internal Review Board on Ethics in Animal Research of the University of São Paulo School of Medicine (process numbers 021/2017, 022/2017, and 1764/2022) and were conducted in accordance with the guidelines for the care and use of laboratory animals established by the US National Research Council.

Male BALB/c mice (*Mus musculus*) at postnatal Day 30 were obtained from the Bioterium Center of the University of São Paulo School of Medicine, Brazil. Animals were housed two per cage in a quiet environment under a 12 h light‐dark cycle at a controlled room temperature of 22°C, with free access to food and water. A total of 12 animals were included in the study, with six mice assigned to the NH and six to NIHL.

### 2.2 Audiological Evaluation

Auditory brainstem responses (ABR) were used to evaluate hearing function using the TDT System 3 RZ6 platform (Tucker Davis Technologies, Alachua, FL, USA). Animals were classified into the NH group, defined by the presence of reproducible ABRs waves between 90‐ and 30‐dB SPL, and the NIHL test group, defined by the absence of detectable waves at 90 dB SPL.

For mice assigned to the NIHL group, ABR recordings were performed before noise exposure to confirm NH and 24 h after exposure to confirm HL. Although five ABR waves are classically described in humans, their correspondence along the auditory pathway differs in rodents (Alvarado et al. 2012). In mice, wave II and the I‐II‐III‐IV complex represent the most consistent parameters for threshold determination and were therefore used in this study.

Animals were anesthetized via intraperitoneal injection of ketamine (80 mg kg^−1^), xylazine (8 mg kg^−1^), and acepromazine (1 mg kg^−1^) (Teuto, Anápolis, GO, Brazil). Body temperature was maintained at approximately 37.5°C using a thermal pad. Recordings were performed inside a Faraday cage to minimize electrical interference. Subdermal electrodes were placed at the vertex (negative), ipsilateral retroauricular region, and contralateral retroauricular region (ground). Electrode impedance was maintained between 1 and 3 kΩ.

Sound stimuli were generated using SigGenRP software (Tucker−Davis Technologies) and delivered via an MF1 Free Field speaker positioned 10 cm from the test ear. Click stimuli were presented in an open‐field configuration. Signals were filtered between 0.3 and 3.0 kHz and averaged across 512 sweeps. Stimuli were initially presented at 90 dB SPL and decreased in 10 dB steps until wave II was no longer detectable. Equipment functionality was verified by testing a normal hearing control animal after noise exposure sessions.

### 2.3 Noise‐Induced Hearing Loss Model

NIHL was induced by exposing P30 mice to broadband white noise at 130 dB SPL for 48 h inside a sound isolation booth. Noise was generated using an audio signal generator (Insight LTDA, Ribeirão Preto, SP, Brazil). Sound intensity gradually increased every 10 min to allow acclimatization and reduce stress.

Four loudspeakers were mounted equidistantly within the booth to ensure uniform sound distribution. Sound field homogeneity was verified using a calibrated sound level meter (DEC 300, Instrutherm, Brazil) at multiple positions within the chamber. Animals were exposed while awake and continuously monitored. Two animals were exposed per session, each housed in a separate cage. Following exposure, animals were returned to their home cages and underwent ABRs testing 24 h later.

### 2.4 Cochlea Dissection and Subsequent Protein Extraction

Animals were euthanized by intraperitoneal injection of ketamine (375 mg kg^−1^) and xylazine (100 mg kg^−1^), followed by decapitation. After antisepsis with 70% ethanol, the skull was opened along the sagittal suture, and the temporal bones were exposed. Cochleae were dissected under a stereomicroscope and transferred to phosphate‐buffered saline on ice.

For proteomic analysis, cochlear tissues were pooled into three biological replicates per condition, each consisting of four cochleae from two animals. Samples were homogenized in 200 µL of lysis buffer containing 8 M urea (Sigma‐Aldrich, St. Louis, MO, USA) and a protease inhibitor cocktail (Sigma‐Aldrich, St. Louis, MO, USA) and then centrifuged at 13,000 rpm for 30 min at 4°C. Supernatants were collected for downstream analysis.

### 2.5 Proteomic Sample Preparation and Nano LC‐MS/MS Analysis

Proteomic analyses were performed at the BIOMASS Core Facility (CEFAP‐USP). Forty micrograms of protein per sample in 8 M urea (Sigma‐Aldrich, Saint Louis, MO, USA) were reduced with 10 mM dithiothreitol (DTT, Sigma‐Aldrich, Saint Louis, MO, USA) and alkylated with 40 mM iodoacetamide (Sigma‐Aldrich, Saint Louis, MO, USA). Samples were diluted and digested overnight with trypsin (Sigma‐Aldrich, Saint Louis, MO, USA) at a 1:50 ratio. We determined peptide concentrations using a Qubit fluorometer (Thermo Fisher Scientific, Waltham, MA, USA).

Peptides were desalted using C18 solid‐phase extraction columns (Thermo Fisher Scientific, Rockford, IL, USA) and dried under vacuum. We conducted nano‐LC‐MS/MS analysis using an EASY‐nLC system coupled to an LTQ Orbitrap Velos mass spectrometer (Thermo Fisher Scientific, San Jose, CA, USA). Peptides were separated on a C18 PicoFrit column using a linear gradient and analyzed in data‐dependent acquisition mode. The 20 most abundant precursor ions were selected for fragmentation by collision‐induced dissociation.

### 2.6 Proteomics Data Processing and Statistical Analysis

Raw MS data were processed using MaxQuant v1.6.1.0 (Cox and Mann 2008) with the Andromeda search engine (Cox et al. 2011; Cox and Mann 2011). MS/MS spectra were searched against the UniProt *Mus musculus* protein database (April 2018 release; 54,747 entries). Database search parameters included: (i) mass tolerance of 4.5 ppm for MS scans and 0.5 Da for MS/MS scans; (ii) trypsin digestion with cleavage at both termini and up to two missed cleavages allowed; (iii) carbamidomethylation of cysteine (57.021 Da) as a fixed modification and oxidation of methionine (15.994 Da) as a variable modification. Protein identification required at least one unique peptide, while protein quantification required a minimum of two ratio counts. A false discovery rate (FDR) of less than 1% was applied at both peptide and protein levels.

Protein quantification was performed using the label‐free quantification (LFQ) algorithm implemented in MaxQuant, which considers both unique and razor peptides. Protein abundance was determined based on LFQ intensity values, representing normalized protein intensities calculated by the MaxQuant software (Cox and Mann 2011).

The MS proteomics data have been deposited in the ProteomeXchange Consortium (Deutsch et al. 2023) via the PRIDE repository (Perez‐Riverol et al. 2024) under the access number PXD058223.

Statistical analysis of proteomic data was conducted using Perseus v1.6.3.1. Proteins identified as reverse hits, potential contaminants, or identified by a single peptide were excluded. LFQ intensity values were log2‐transformed, and proteins without valid values were removed. Protein abundances were estimated based on the summed intensities of unique and razor peptides across runs, followed by normalization to account for intersample variation, ensuring comparability across conditions (Tyanova et al. 2016). Normalized and log2‐transformed LFQ values were further standardized using *Z*‐scores and visualized in heatmaps.

For differential abundance analysis, only proteins with valid LFQ values in at least two biological replicates of at least one condition (NH and NIHL) were considered. Statistical comparisons between NH and NIHL groups were performed using an unpaired two‐sided Student's *t*‐test implemented in Perseus. Statistical significance was defined using a *p* ≤ 0.05, together with an absolute log2 fold change (|log2FC |) ≥ 0.2 and an absolute t‐test statistic (|t |) ≥ 2.5. Fold change (FC) values were calculated as the difference between the mean log2‐transformed LFQ intensities of the two conditions, with positive values indicating higher abundance in NIHL and negative values indicating lower abundance in NIHL (Tyanova et al. 2016). The *t*‐test statistics and corresponding *p* values were used for graphical representation.

Proteins meeting the criteria of *p* ≤ 0.05, |log2FC | ≥ 0.2, and |*t* | ≥ 2.5 were classified as differentially abundant proteins (DAPs). Volcano plots and bar charts were generated using Perseus, GraphPad Prism, or SRplot. Additionally, proteins detected in at least two biological replicates of one condition but completely absent in the other condition were classified as condition‐specific proteins and considered separately from the statistically tested proteins. These proteins were included in downstream bioinformatics and omics data reuse analyses.

Because of the limited amount of protein obtainable from individual mouse cochleae, cochlear tissues were pooled prior to MS analysis to ensure sufficient material for proteomic profiling. As the resulting bulk proteomic data do not provide cell‐type resolution, the identified DAPs were subsequently integrated with publicly available cochlear single‐cell transcriptomic datasets to provide complementary evidence regarding their cell‐type distribution.

DAPs were subsequently subjected to an integrative bioinformatics workflow combining complementary analytical approaches. Mouse proteins were investigated through functional enrichment analyses, pathway analysis, protein–protein interaction (PPI) network analysis, comparison with an independent cochlear proteomic data set, integration with publicly available cochlear single‐cell transcriptomic datasets, and mouse phenotype annotation. Mouse genes were then converted to their corresponding human orthologs. These orthologs were further analyzed through PPI network analysis, mapping to unresolved hereditary hearing loss loci, human phenotype and disease annotations, and translational target prioritization. The combined results of these complementary analyses were integrated to support the biological interpretation and prioritization of DAPs.

### 2.7 Protein Class, Gene Ontology, and Pathways Over‐Representation Analysis

Protein class analysis of the Global proteome (combined NH and NIHL) was performed using the PANTHER database (http://www.pantherdb.org).

Over‐representation analysis (ORA) of Gene Ontology (GO) terms and pathways (KEGG, Reactome, PANTHER, and WikiPathways) was carried out with WebGestalt (https://www.webgestalt.org/), based on UniProt accession numbers from NH, NIHL, and NH versus NIHL DAP datasets. The protein‐coding genome was used as the reference set for enrichment analysis. Multiple testing correction was applied using the Benjamini–Hochberg procedure, with significance thresholds set at *p* < 0.05 and FDR < 0.05. Redundant GO terms were minimized to improve interpretability.

For the NH and NIHL proteomes analyzed independently, the weighted set cover algorithm implemented in WebGestalt was applied to identify the most representative and informative GO terms across biological processes (BP), cellular components (CC), and molecular functions (MF). In contrast, for the DAPs, only redundancy reduction was applied, without the use of weighted set cover. The weighted set cover algorithm does not remove true enrichments; rather, it selects a representative subset of nonredundant GO terms based on statistical significance and gene coverage. Consequently, the absence of a specific GO term in the reduced output does not indicate a lack of enrichment in the dataset but instead reflects prioritization of terms considered more informative. Therefore, comparisons between NH and NIHL enrichment profiles should be interpreted as reflecting the relative representativity of functional categories rather than their strict presence or absence.

Only enriched categories showing biological coherence with complementary analyses were considered during downstream interpretation. Enrichment significance alone was not considered sufficient evidence of biological relevance.

### 2.8 Single‐Cell Transcriptomic Mapping of Cochlear Proteomic Data

To investigate the cell‐type distribution and transcriptional context of proteins identified in the cochlear proteome, the proteomic datasets were integrated with the mouse cochlear single‐cell RNA sequencing (scRNA‐seq) atlas published by Jean et al. (2023). This reference dataset comprises more than 120,000 cells representing 25 cochlear cell types across three developmental stages (P8, P12, and P20), capturing the transition from prehearing to auditory maturity. For the present analysis, the P20 data set was used as the reference for the mature cochlea. For downstream analyses, individual cell types were grouped into seven functional categories: circulating cells (B cells, macrophages, monocytes, neutrophils, NK cells, T cells, mast cells, erythrocytes); lateral wall (endothelial cells, pericytes, basal, intermediate, and marginal strial cells, fibrocytes, spindle cells, root cells); supporting cells (Claudius, inner sulcus, and outer sulcus cells; Deiters' cells; pillar cells; interdental cells); hair cells (inner hair cells); neurons (type I and type II); glial cells (precursors, satellite glia, Schwann cells); and surrounding structures (SS1, SS2, Reissner's membrane cells, scala vestibuli border cells, and tympanic border cells).

Protein identifiers from each proteomic dataset (NH, NIHL, and DAPs) were mapped to their corresponding gene symbols and evaluated using the P20 gene‐rank tables from the mature cochlear single‐cell atlas. Genes present in the rank tables were considered detectable, and for each data set and cochlear cell group, both the total number and the proportion of detectable genes were calculated. No additional normalization was required because the rank tables were already preordered by gene expression within each cell type.

In a second analysis, DAPs were used to estimate relative transcript expression across cochlear cell types. For each gene, an expression percentile (P) was calculated for each cell type using the formula: P = (1 − [gene rank]/[total number of genes in the cell type]) × 100. The Top X% metric was then defined as 100 − P, representing the proportion of genes in the cell type with equal or lower expression. For example, a DAP with a Top X% value < 20% is among the top 20% most highly expressed genes in that cell type. This analysis enabled a cell‐type‐resolved assessment of the transcriptional context of proteins detected in the proteome and supported the identification of potentially enriched cell‐type associations of DAPs.

### 2.9 Auditory Phenotype Ontology, Disease Mapping of Hearing‐Related Genes, and Deafness Locus Annotation

The Monarch Initiative (https://monarchinitiative.org/) and the Human Phenotype Ontology (https://hpo.jax.org/) were used to identify phenotype terms related to the inner ear structure and auditory function. The Monarch Initiative integrates gene, variants, disease, and phenotype information across multiple species, whereas the Human Phenotype Ontology (HPO) provides standardized annotations of human phenotypic abnormalities. Together, these resources were used to investigate genotype–phenotype relationships relevant to HL. DAPs were mapped to phenotype terms associated with HL, auditory dysfunction, auditory morphology, inner ear pathology, and cochlear repair. To assess the clinical relevance of these genes, the Online Mendelian Inheritance in Man (OMIM; https://www.omim.org/), ORPHANET (https://www.orpha.net/), and MONDO (https://mondo.monarchinitiative.org/) databases were queried to determine whether the corresponding human genes were associated with syndromic or nonsyndromic forms of HL.

Mouse genes corresponding to the DAPs were converted to their human orthologs using Ensembl Compare. Human genomic coordinates were retrieved from Ensembl and RefSeq and compared with the genomic intervals of hereditary HL (DFN) loci curated in the Hereditary Hearing Loss Homepage (Walls et al. 2025). For each human ortholog, we determined whether the gene was located within the mapped DFN interval or outside its boundaries. For genes positioned outside the interval, the genomic distance to the nearest flanking marker was calculated to assess positional proximity. Original publications describing each DFN locus were consulted to confirm locus boundaries and associated markers. Information on known deafness genes and cochlear gene expression was additionally obtained from the SHIELD database (https://shield.hms.harvard.edu/). This orthology‐guided positional mapping strategy was designed to prioritize DAP‐associated genes located within unresolved hereditary HL loci as positional candidate genes or candidate modifiers. The analysis was intended to support candidate prioritization rather than to identify causative genes for individual DFN loci.

### 2.10 Druggability Analysis

DAPs were classified by druggability using the Pharos database (https://pharos.nih.gov/), which organizes targets according to the Illuminating the Druggable Genome (IDG) framework. Targets were categorized into four tiers: Tclin, proteins with at least one approved therapeutic drug; Tchem, proteins with at least one high‐potency small‐molecule ligand (activity cutoff < 30 nM); Tbio, proteins lacking approved drugs or high‐affinity ligands but supported by biological evidence such as GO annotations and disease associations; and Tdark, proteins for which limited functional, chemical, or biological information is currently available.

Druggability information was incorporated as one component of the integrative bioinformatics workflow to support the translational prioritization of DAPs.

### 2.11 Comparison With an External Proteomic Dataset

To assess the external consistency of the proteomic findings, the identified DAPs were compared with an independent cochlear proteomic dataset generated using a distinct experimental design. Specifically, our DAPs were compared with the dataset reported by Jongkamonwiwat et al. (2020), which analyzed cochlear protein expression in adult mice exposed to high‐ and very‐high‐intensity noise. Proteomic results were extracted from the published supplementary tables and only proteins reported as significantly differentially abundant (*p* < 0.05; |FC | ≥ 1.5) were included. Overlapping proteins between the two data sets were subsequently identified and used to support the robustness of the proteomic findings.

Protein identifiers were matched between data sets to identify overlapping proteins. Overlapping proteins between the two data sets were subsequently identified and used to provide independent comparative evidence supporting the consistency of the proteomic findings.

This comparative analysis represented an independent line of evidence within the integrative bioinformatics workflow used to prioritize biologically relevant proteins.

### 2.12 PPI Network

To explore molecular interactions among DAPs, PPI network analysis was performed using the STRING database (https://string-db.org). Mouse and their corresponding human orthologs were analyzed independently to assess interaction patterns within each species and identify conserved network relationships. *Mus musculus* gene symbols corresponding to proteins identified in the NH versus NIHL comparison were used as input. Interaction networks were generated using either the complete DAP list (*n* = 80) or selected protein subsets, depending on the specific biological hypothesis under investigation.

STRING interaction scores represent confidence in a predicted protein–protein association and range from 0 to 1, with higher scores indicating greater confidence. For network construction and visualization, associations with combined scores ≥ 0.4 were considered, encompassing medium‐ (≥ 0.4), high‐ (≥ 0.7), and highest‐confidence (≥ 0.9) associations according to STRING confidence categories. Higher‐scoring associations were highlighted in the interpretation as stronger evidence supporting the corresponding protein associations. Network analyses were incorporated into the integrative bioinformatics workflow to support the functional interpretation and prioritization of DAPs.

## 3 Results

### 3.1 Comprehensive Cochlear Proteomic Landscape in Normal and Noise‐Exposed Mice

To establish a comprehensive baseline for cochlear function and pathology, global proteomic profiling was performed on cochlear samples from NH and NIHL mice. In total, 1742 proteins were identified: 1572 in at least one NH replicate and 1605 in the NIHL group. Most proteins (1435) were shared between the two groups, whereas 137 were unique to NH and 170 were unique to NIHL (Figure 1A,B; Supporting Information S1: Table 1A).

**Figure 1:**
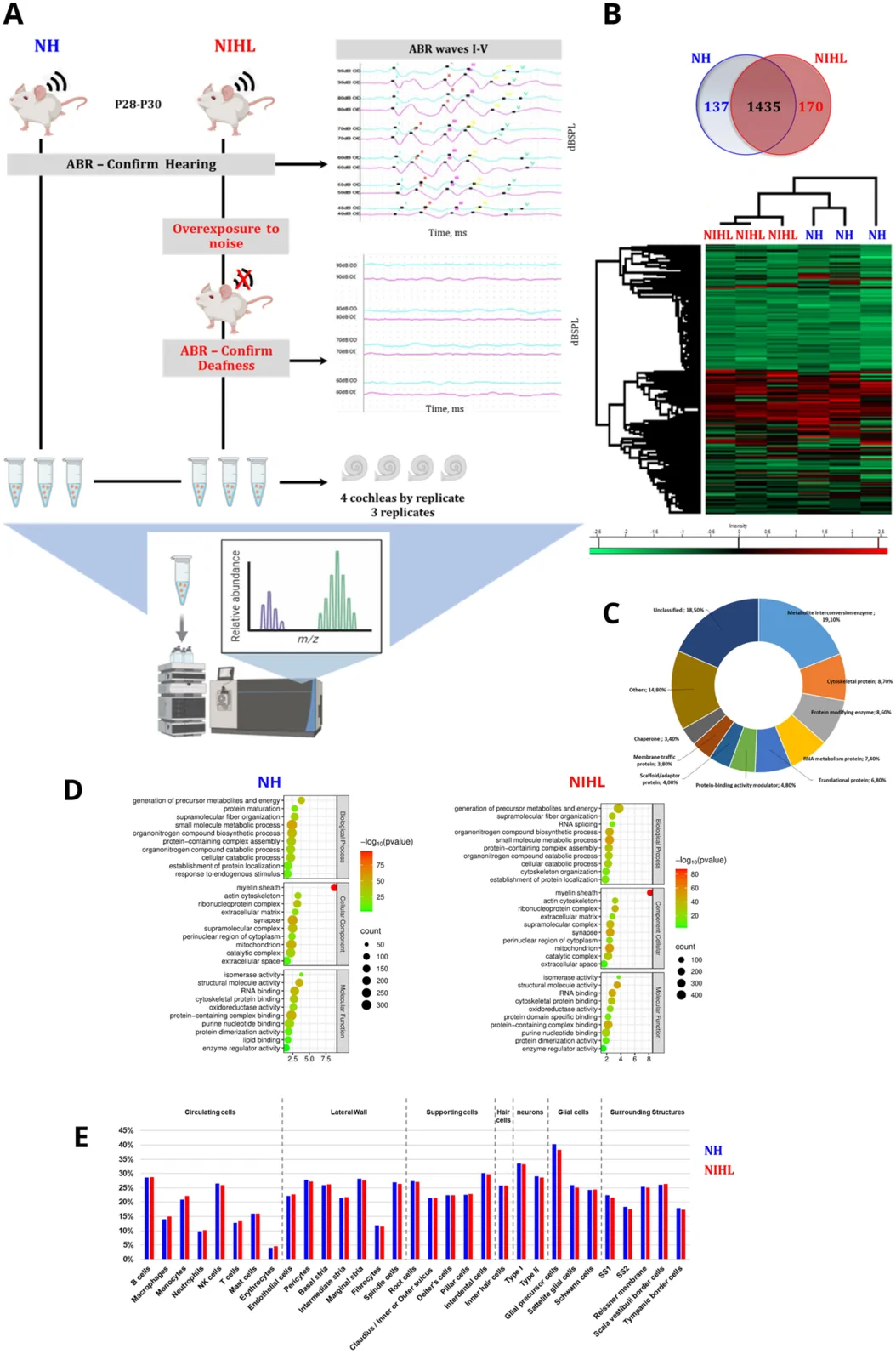
Workflow for identification and characterization of the murine cochlear proteome under NH and NIHL. (A) Quantitative MS analysis was performed in triplicate using pooled cochleae from male Balb/c mice: young adults with NH (P28–P30) and age‐matched mice with NIHL. Each replicate comprised two animals (four cochleae), totaling 12 mice (6 NH and 6 NIHL). NIHL was induced by white noise (130 dB SPL). Auditory function was assessed by ABR: NH was confirmed prior to noise exposure, and NIHL was verified within 24 h postexposure by the absence of response waves up to 90 dB SPL. (B) Global cochlear proteome profiling. Venn diagram showing the number of proteins identified in NH and NIHL conditions, alongside a Heatmap of log_2_‐transformed LFQ intensities after *z*‐score normalization. (C) Protein class distribution of the 1742 identified proteins based on PANTHER classification. (D) GO over‐representation analysis of biological process (BP), cellular component (CC), and molecular function (MF) categories for NH (red) and NIHL (blue) proteomes. Results are shown as bubble plots, with fold enrichment on the *x*‐axis and enriched GO terms on the *y*‐axis; bubble size reflects the number of proteins, and color denotes –log_10_ adjusted *p* values. Redundant GO terms were reduced using a weighted set‐cover algorithm. (E) Distribution (%) of protein‐coding genes across major cochlear cell types—including hair cells, spiral ganglion neurons, glial cells, supporting cells, lateral wall cells, circulating cells, and surrounding structures—mapped to the P20 mouse cochlear single‐cell RNA‐seq atlas (Jean et al. 2023).

Data quality and sample consistency were evaluated using protein intensity distributions and unsupervised clustering analyses. Heatmap visualization following normalization and *Z*‐score transformation demonstrated clustering by experimental condition, supporting the consistency of the proteomic profiles (Figure 1B).

Forty‐eight protein entries with multiple identifiers (Supporting Information S1: Table 1B) were grouped to account for isoforms, redundancies, or shared peptide sequences, a common feature in proteomic data sets, thereby avoiding overestimation of unique protein counts.

Functional classification of the total cochlear proteome, combining NH and NIHL samples, was performed using the PANTHER system and revealed a broad diversity of protein classes (Figure 1C; Supporting Information S1: Table 1C). The most abundant categories were metabolite interconversion enzymes (19.1%), cytoskeletal proteins (8.7%), and protein‐modifying enzymes (8.6%). Additional protein classes included RNA metabolism proteins (7.4%), translational proteins (6.8%), protein‐binding activity modulators (4.8%), scaffold/adaptor proteins (4.0%), membrane trafficking proteins (3.8%), and chaperones (3.4%). Proteins categorized as “others” accounted for 14.8% of the dataset, while 18.5% were annotated as “unclassified,” corresponding to proteins without functional categorization in the PANTHER system.

Among the 1742 proteins identified, 25 were encoded by orthologous genes associated with or strongly implicated in human deafness, including *ACTG1, AIFM1, ATP6V1B2, CEACAM16, COCH, COL11A1, COL11A2, COL2A1, COL9A1, COL9A2, CRYM, EPS8L2, HSD17B4, KARS, MYH1, MYH9, PNPT1, PRPS1, RDX, SERPINB6, SLC12A2, TECTA, TJP2, TNC*, and *TRIOBP* (Walls et al. 2025). The presence of these deafness‐associated proteins further supports the biological relevance of the identified cochlear proteome for studying hearing‐related molecular processes (Supporting Information S1: Table 1D).

### 3.2 Functional Similarity and Distinctive Functional Features of the NH and NIHL Cochlear Proteomes

Functional enrichment analysis of the NH and NIHL cochlear proteomes was performed with weighted set cover reduction to identify non‐redundant, representative GO terms (Figure 1D; Supporting Information S1: Table 1E).

A high degree of functional similarity was observed between the NH and NIHL proteomes. The shared functional profile included biological process terms related to energy metabolism (generation of precursor metabolites and energy), structural organization (supramolecular fiber organization and cytoskeleton organization), and protein synthesis and turnover (organonitrogen compound biosynthetic and catabolic processes, and protein‐containing complex assembly). Similar patterns were observed across molecular function and cellular component categories, including enrichment for RNA binding, structural molecule activity, and cytoskeletal protein binding, as well as proteins associated with the myelin sheath, actin cytoskeleton, synapse, and mitochondrion (Figure 1D).

Despite this overall functional similarity, weighted set cover reduction highlighted differences in functional categories retained as representative of each condition. Terms related to protein maturation (GO:0051604) and response to endogenous stimulus (GO:0009719) were retained for the NH proteome, whereas RNA splicing (GO:0008380) and cytoskeleton organization (GO:0007010) were retained for the NIHL condition. These differences indicate distinct functional features in the reduced enrichment profiles of the two conditions.

To further investigate the cellular context, the cochlear protein‐coding genes were mapped to a published single‐cell RNA sequencing atlas of the P20 mouse cochlea (Jean et al. 2023). Of the 1,742 protein‐coding genes identified in the proteomic dataset, 1,635 were successfully assigned to at least one of seven major cochlear cell groups: circulating cells, lateral wall, supporting cells, hair cells, neurons, glial cells, and surrounding structures. This analysis demonstrated broad representation across cochlear cell groups, with the highest representation observed in glial cells, supporting cells, and neurons (Figure 1E; Supporting Information S1: Table 1F). The remaining 107 genes were not represented in the P20 transcriptomic atlas. Their absence from the atlas may reflect differences in developmental stage, low or spatially restricted transcript expression, posttranscriptional regulation, or limitations in single‐cell transcriptomic detection (Supporting Information S1: Table 1E).

### 3.3 Identification of DAPs in NIHL Cochleae

Comparative proteomic analysis between NH and NIHL cochleae identified proteins showing differences in abundance between the two conditions. From the filtered data set, 1245 proteins with valid LFQ values in at least two biological replicates of at least one condition were considered for differential abundance analysis. Statistical comparison using an unpaired two‐sided Student's *t*‐test identified 50 proteins meeting the predefined criteria of *p* ≤ 0.05, |log_2_FC | ≥ 0.2, and |t | ≥ 2.5 (Supporting Information S2: Table 2A).

Following exclusion of two hemoglobin subunits (HBA/P01942 and HBB2‐B2/P02089), 48 proteins remained. Among these, two entries corresponding to Serpina1 isoforms (SERPINA1B/P22599 and SERPINA1E/Q00898) were consolidated because they could not be reliably distinguished based on the detected peptides. This resulted in a curated set of 49 quantitatively defined DAPs, comprising 23 proteins with higher abundance and 26 with lower abundance in NIHL compared with NH (Figure 2A,B and Supporting Information S2: Table 2A).

**Figure 2:**
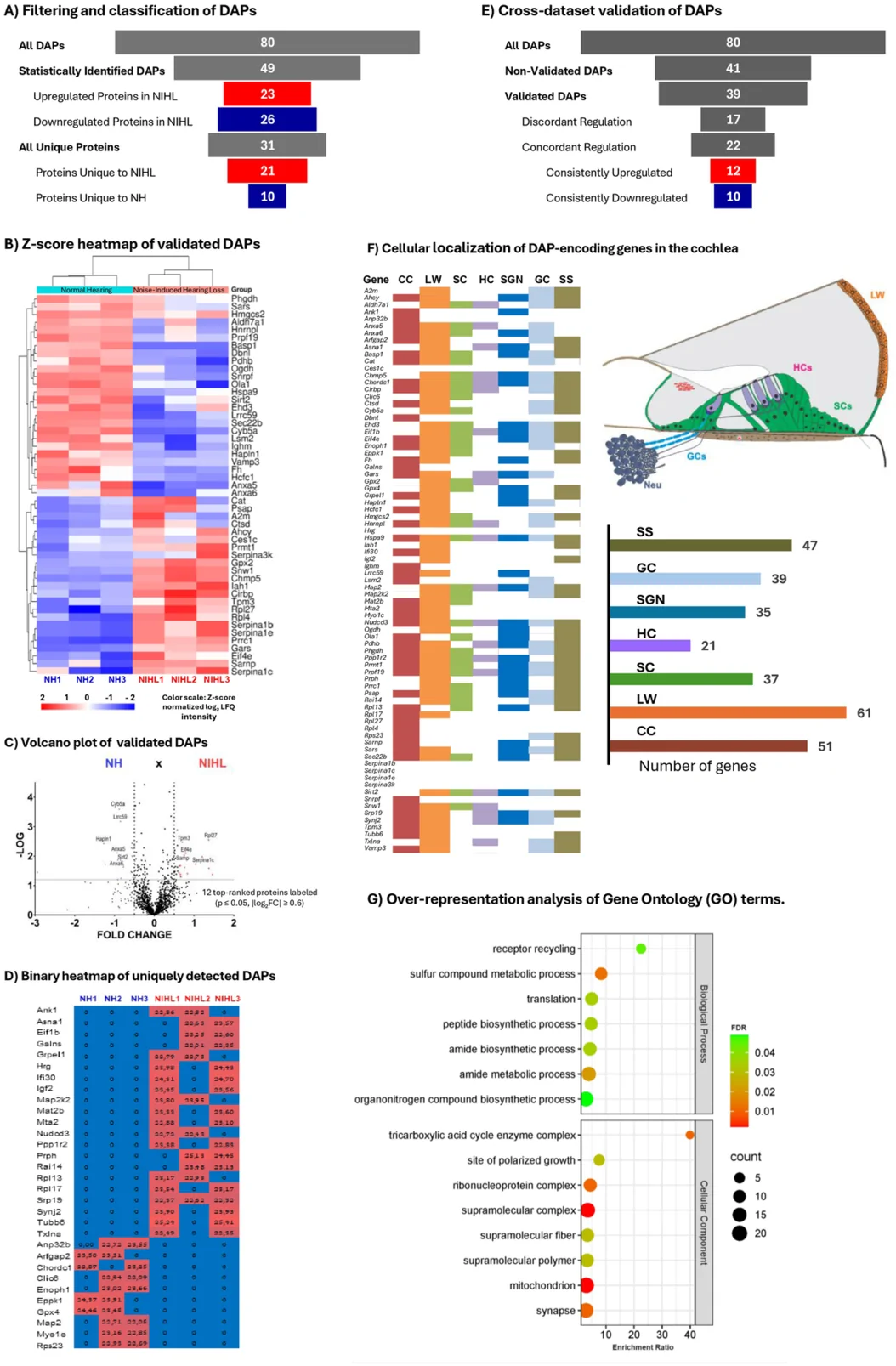
DAPs in NH and NIHL cochleae and in silico characterization. (A–G) summarize the identification, classification, validation, cellular distribution, and functional enrichment of DAPs in NH and NIHL cochleae. Complete protein quantification, statistical analyses, and enrichment results are provided in Supporting Information S1: Table 2A–C, and cell‐type mapping data in Supporting Information S3: Table 3. (A) Funnel‐style overview of DAP identification and filtering. Eighty proteins were initially detected, of which 49 met statistical significance criteria (*p* ≤ 0.05, |log_2_FC| ≥ 0.2, and |t | ≥ 2.5). Among these, 23 proteins were upregulated (red) and 26 downregulated (blue) in NIHL. In addition, 31 proteins were uniquely detected in a condition‐specific manner, including 21 exclusive to NIHL and 10 exclusive to NH. (B) Heatmap showing hierarchical clustering of the 49 statistically validated DAPs (*p* ≤ 0.05, |log_2_FC| ≥ 0.2). Values represent Z‐score–normalized log_2_ LFQ intensities across three biological replicates per group (NH1–NH3 and NIHL1–NIHL3). Red and blue indicate relative upregulation and downregulation in NIHL, respectively. (C) Volcano plot depicting differential protein abundance between NH and NIHL cochleae. Each point represents one quantified protein (*n* = 49). Proteins meeting significance thresholds (*p* ≤ 0.05, |log_2_FC| ≥ 0.6) are highlighted in red (upregulated) or blue (downregulated), with the eleven most significantly altered proteins labeled. Non‐significant proteins are shown in black. (D) Presence/absence Heatmap of uniquely detected proteins in NH and NIHL cochleae. The binary Heatmap displays 31 proteins detected exclusively in one condition (10 in NH, 21 in NIHL) across individual biological replicates (NH1–NH3, NIHL1–NIHL3). Blue denotes absence (undetected; value = 0), and red indicates presence (detected proteins). (E) Independent validation of DAPs using a published cochlear noise‐response dataset (Jongkamonwiwat et al. 2020). Bar plots summarize validation status and concordance of expression trends for all 80 DAPs. Thirty‐nine proteins were also differentially expressed in the external dataset, of which 22 showed concordant regulation (12 upregulated and 10 downregulated in NIHL), while 17 exhibited discordant trends. These findings support the reproducibility of a subset of NIHL‐associated proteomic changes. (F) Single‐cell expression mapping of 74 DAP‐associated transcripts across seven major cochlear cell populations: lateral wall (LW), supporting cells (SC), hair cells (HC), spiral ganglion neurons (SGN), glial cells (GC), mesenchymal/surrounding structures (SS), and immune/circulating cells (CC), based on the mouse cochlear single‐cell RNA‐seq atlas (Jean et al. 2023). The Heatmap indicates transcript presence (colored) or absence (white) within each cell type, while an accompanying bar chart quantifies the number of detected genes per population. Colors correspond to cell‐type codes used throughout the figure. A schematic transverse cochlear section illustrates the anatomical localization of each population. (G) Bubble plot showing significantly enriched GO categories (biological process and cellular component) among DAPs (*p* ≤ 2 × 10^−5^, FDR ≤ 0.05). Bubble size reflects the number of mapped proteins, and color intensity denotes adjusted FDR values. Full enrichment statistics are provided in Supporting Information S2: Table 2C.

The proteins showing the largest abundance differences (*p* ≤ 0.05 and |log_2_FC | ≥ 0.6) included six proteins with lower abundance in NIHL: HAPLN1 (Q9QUP5), SIRT2 (Q8VDQ8), ANXA5 (P48036), ANXA6 (P14824), CYB5A (P56395), and LRRC59 (Q922Q8); and five proteins with higher abundance in NIHL: TPM3 (P21107), SARNP (Q9D1J3), EIF4E (P63073), SERPINA1C (Q00896), and RPL27 (P61358) (Figure 2C and Supporting Information S2: Table 2A).

In addition to the statistically tested proteins, condition‐specific proteins were identified separately based on their detection pattern. Proteins detected in at least two biological replicates of one condition and completely absent in the other condition were classified as condition‐specific proteins. This analysis identified 31 proteins, including 21 detected exclusively in NIHL and 10 detected exclusively in NH (Figure 2A,D; Supporting Information S2: Table 2A). Because these proteins were defined by condition‐specific detection rather than by statistical testing, they were considered separately from the quantitatively defined DAPs.

The 49 quantitatively defined DAPs and 31 condition‐specific proteins were subsequently combined to generate the 80‐protein set used for downstream integrative analyses. This set comprised 44 proteins with higher abundance or exclusive detection in NIHL and 36 proteins with lower abundance or exclusive detection in NH (Figure 2A; Supporting Information S2: Table 2A).

### 3.4 Cross‐Study Comparison Reveals a Shared Noise‐Responsive Cochlear Proteomic Signature

To assess the concordance of the NIHL‐associated proteomic signature across independent data sets, the 80‐protein data set identified in the present study was compared with an independent cochlear proteomic dataset generated following noise exposure by Jongkamonwiwat et al. (2020). Regulation status, FC, and concordance across the two studies are detailed in Supporting Information S2: Table 2B.

This cross‐study comparison identified 39 of the 80 proteins (48.8%) detected in the present study as dysregulated in the independent dataset following exposure to 94‐ or 105‐dB SPL noise (Figure 2D; Supporting Information S2: Table 2B). Among these overlapping proteins, 22 showed concordant directional changes in at least one of the two noise‐exposure conditions, indicating that a subset of the proteins identified in the present study showed similar directional responses in an independent noise‐exposure dataset.

Ten proteins showed consistent downregulation across studies. ANXA6 (P14824), CYB5A (P56395), HAPLN1 (Q9QUP5), MAP2 (P20357), and VAMP3 (P63024) were downregulated at both 94‐ and 105‐dB SPL; ANXA5 (P48036) was concordantly downregulated at 105 dB SPL, whereas BASP1 (Q91XV3), HSPA9 (P38647), and PRPF19 (Q99KP6) showed concordant downregulation at 94 dB SPL. CHORDC1 (Q9D1P4) was downregulated in the present dataset but exhibited different directional responses depending on noise intensity in the independent study.

Twelve proteins showed concordant upregulation across the two datasets. NUDCD3 (Q8R1N4), PRRC1 (Q3UPH1), and SNW1 (Q9CSN1) were upregulated at both noise intensities; GALNS (Q571E4), HRG (Q9ESB3), IAH1 (Q9DB29), MAT2B (Q99LB6), and PRPH (P15331) showed concordant upregulation at 105 dB SPL; and CIRBP (P60824), GARS (Q9CZD3), IGF2 (P09535), and PRMT1 (Q9JIF0) showed concordant upregulation at 94 dB SPL.

The remaining 17 overlapping proteins showed discordant directional changes between the data sets (Supporting Information S2: Table 2B).

The cross‑study comparison identified a reproducible core of noise‑responsive cochlear proteins, despite variability in individual protein responses across noise intensities. These independent findings reinforce the robustness of selected protein‑level changes and justify their prioritization in downstream functional and cellular analyses.

### 3.5 Transcriptional Landscape of NIHL‐Associated Proteins Across Cochlear Cell Types

To contextualize the cellular distribution of genes encoding the NIHL‐associated proteins, we mapped the corresponding transcripts onto a single‐cell RNA‐sequencing atlas of the mature, normal‐hearing mouse cochlea (P20) (Jean et al. 2023). This reference data set represents the basal transcriptional landscape of the cochlea under physiological conditions and therefore provides a cell‐type framework for assessing where genes corresponding to the proteomic findings are transcriptionally active in the absence of noise exposure.

Of the 80 proteins identified in the NIHL‐associated proteomic signature, transcripts corresponding to 74 genes were detected across seven major cochlear cell populations: lateral wall, circulating or immune cells, supporting cells, spiral ganglion neurons, hair cells, glial cells, and surrounding mesenchymal structures. Six genes (*Ces1c, Hrg, Serpina1b, Serpina1c, Serpina1e*, and *Serpina3k*) were not detected in the reference atlas, potentially reflecting low basal transcript abundance, condition‐specific expression, or technical limitations of the data set (Figure 2E; Supporting Information S3: Table 3). The complete cell‐type distribution and percentile‐based expression rankings for the DAP‐encoding genes are provided in Supporting Information S3: Table 3. Most DAP‐encoding genes showed broad transcriptional activity across multiple cochlear cell populations. Several exhibited near‐ubiquitous expressions, including *Chordc1, Hspa9*, and *Prpf19*, whereas others showed more restricted patterns, such as *Ighm* and *Anp32b* in immune‐related clusters.

Across the reference atlas, percentile‐based analysis showed that DAP‐encoding genes were represented across diverse cochlear cell populations, including lateral wall, supporting, immune, neuronal, glial, and sensory hair cell populations (Figure 2E; Supporting Information S3: Table 3). Within lateral wall populations, several genes ranked among the most highly expressed transcripts in specific subpopulations. *Gpx2* ranked within the top 0.4% of transcripts in root cells, while *Ehd3* showed high expression across multiple stria vascularis subtypes, ranking within the top 1.1% and 2.2% in marginal and basal stria, respectively. *Eppk1* and *Rai14* also showed high expression in basal stria, spindle cells, root cells, and endothelial cells. Additional genes, including *A2m, Anxa5, Cirbp*, and *Sirt2*, showed substantial expression across distinct lateral wall subtypes (Supporting Information S3: Table 3). Supporting cell populations similarly showed prominent representation of DAP‐encoding transcripts. *Gpx2* ranked within the top 0.5% of transcripts in Deiters and pillar cells, while *Clic6* and *Anxa5* showed relatively high expression in outer sulcus, interdental, and Deiters cells.

Immune cell clusters were characterized by high expression of *Ighm*, which ranked within the top 0.6% of transcripts in B cells, as well as *Psap* and *Ifi30* in macrophage and monocyte populations (Supporting Information S3: Table 3). These patterns indicate that several proteins altered in the whole‐cochlear proteome are encoded by genes that are transcriptionally active in immune and supporting compartments under basal conditions.

A smaller subset of DAP‐encoding genes showed prominent expression in neuronal and glial populations, including *Map2* and *Prph* in spiral ganglion neurons and *Hmgcs2* in Schwann cells. In contrast, relatively few DAP‐encoding genes ranked highly in sensory hair cells, with *Gpx2* and *Anxa5* representing notable examples (Figure 2E and Supporting Information S3: Table 3).

Importantly, this transcriptional mapping does not establish the cellular origin of the observed protein abundance changes, because the proteomic measurements were generated from whole‐cochlear lysates. Likewise, because the reference atlas represents NH cochleae in the absence of noise exposure, the observed transcriptional profiles cannot be assumed to reflect cell‐state changes induced by acoustic trauma. Rather, this analysis provides a cellular framework showing that genes corresponding to the NIHL‐associated proteomic signature were represented among highly expressed transcripts across diverse cochlear cell populations under basal conditions, including structural, supporting, lateral wall, immune, neuronal, glial, and sensory hair cell populations. This framework provides a basis for generating hypotheses regarding cell‐type‐specific contributions to the molecular response to noise and for prioritizing candidates for future spatial and functional validation.

### 3.6 Functional Enrichment Highlights Metabolic, Biosynthetic, and Protein‐Handling Processes Associated With NIHL

To characterize the biological processes represented within the 80‐protein NIHL‐associated signature, we performed functional enrichment analysis across GO Biological Process and Cellular Component categories and KEGG pathways. Significant enrichment was observed across multiple functional categories (FDR < 0.05; Figure 2F; Supporting Information S2: Table 2C). Rather than being concentrated in a single functional category, the enriched proteins represented a range of metabolic, biosynthetic, protein synthesis, and cellular organization processes.

Among GO Biological Process terms, sulfur compound metabolic process was significantly enriched (9 proteins; enrichment ratio = 8.25; FDR = 0.0123), as were amide metabolic process (15 proteins; enrichment ratio = 3.90; FDR = 0.0211), translation (11 proteins; enrichment ratio = 4.87; FDR = 0.0349), and amide and peptide biosynthetic processes (12 and 11 proteins, respectively; FDR = 0.0349). The enrichment of sulfur compound metabolism is consistent with the presence of proteins involved in sulfur‐containing metabolic pathways, whereas the enrichment of translation and peptidation of protein‐synthesis‐related proteins within the NIHL‐associated signature.

GO Cellular Component analysis also identified enrichment for mitochondria (20 proteins; enrichment ratio = 3.04; FDR = 0.00229), the tricarboxylic acid cycle enzyme complex (3 proteins; enrichment ratio = 39.86; FDR = 0.01295), ribonucleoprotein complexes (11 proteins; enrichment ratio = 4.42; FDR = 0.0120), synaptic regions (17 proteins; enrichment ratio = 2.93; FDR = 0.0120), and sites of polarized growth (6 proteins; enrichment ratio = 7.61; FDR = 0.0263) (Figure 2F; Supporting Information S2: Table 2). The enrichment of mitochondrial and tricarboxylic acid cycle components is consistent with the representation of proteins involved in cellular energy metabolism, while synaptic regions and sites of polarized growth encompass structural and functional features relevant to cochlear cells.

KEGG pathway analysis identified cysteine and methionine metabolism (mmu00270) as the most significantly enriched pathway (Figure 2F; Supplementary Table 2 C). This pathway provides a metabolic context for the enrichment of sulfur compound metabolism observed in the GO analysis and encompasses processes related to sulfur amino acid metabolism and methyl‐group metabolism.

The complete list of enriched GO terms and KEGG pathways, together with the proteins contributing to each category and their enrichment statistics, is provided in Supporting Information S2: Table 2.

### 3.7 In‐Silico Prioritization Integrates Genetic, Phenotypic, and Pharmacological Evidence

To further contextualize the 80‐protein NIHL‐associated signature, we integrated complementary human and mouse annotation resources covering hereditary hearing loss loci, auditory phenotypes, human disease associations, and pharmacological tractability. This multilayered approach identified proteins with convergent evidence linking them to auditory function, disease phenotypes, or genomic regions associated with HL, thereby providing complementary biological and genetic context for the NIHL‐associated proteome.

Fifteen proteins in the NIHL‐associated signature corresponded to mouse genes whose human orthologs could be positioned relative to unresolved hereditary hearing loss (DFN) loci based on orthology‐guided positional mapping. These genes were prioritized as positional candidates for further investigation within unresolved DFN regions rather than as established causal genes. The positional relationship of each ortholog to the corresponding DFN interval, including whether the gene was located within or outside the mapped interval, its genomic proximity to flanking markers, and the publications defining each locus, is detailed in Supporting Information S2: Table 2D.

Proteins in the NIHL‐associated signature were also cross‐referenced with curated auditory phenotype annotations from human and mouse resources. Sixteen proteins had documented associations with hearing function or ear morphology (Supporting Information S2: Table 2E). In addition, 14 proteins were implicated in human syndromic disorders in which hearing impairment or other ear‐related abnormalities are reported clinical features (Supporting Information S2: Table 2F).

The auditory phenotype and human disease annotation layers identified a combined set of 18 proteins supported by at least one of these complementary lines of evidence, which were prioritized in the integrated candidate summary (Table 1). These proteins were CAT, CTSD, FH, GALNS, GPX4, HAPLN1, HCFC1, HSPA9, IGF2, IGHM, MAP2K2, MYO1C, PHGDH, PSAP, RPS23, SRP19, SYNJ2, and TPM3.

**Table 1:** Auditory phenotypes, disease associations, and supporting annotations of selected differentially abundant proteins.

Protein ID (mouse)	Gene mouse	Gene Human	Our MS | regulated in NIHL	External data set noise exposure | regulated	Unresolved DFN locus	Ontology phenotype		Human disease	
						Human	Mammals	Name syndrome or disease	Term name
P24270	*Cat*	*CAT*	UP	—	—	Sensorineural HL and other inner ear abnormalities	‐‐	Acatalasemia MONDO:0013571 ORPHA:926	Age‐associated sensorineural HL
P18242	*Ctsd*	*CTSD*	UP	DOWN	—	Abnormal morphology and positioning of the external ear	Neuroauditory abnormalities encompassing HL and altered auditory brainstem responses	Ceroid lipofuscinosis, neuronal, 10 MONDO:0012414 OMIM:610127	Low‐set ears
P97807	*Fh*	*FH*	DOWN	—	—	Structural and functional abnormalities of the auditory system, including HI and tinnitus	—	Hereditary pheochromocytoma‐paraganglioma MONDO:0017366 ORPHA:29072	Conductive HI
Q571E4	*Galns*	*GALNS*	UP	UP	—	HI and structural or physiological abnormalities of the ear	—	Mucopolysaccharidosis IVA MONDO:0009659 OMIM:253000	HI
O70325	*Gpx4*	*GPX4*	DOWN	—	DFNB72	Abnormal morphology and positioning of the external ear	—	Spondylometaphyseal dysplasia, Sedaghatian type MONDO:0009593 OMIM:250220	Posteriorly rotated ears
Q9QUP5	*Hapln1*	*HAPLN1*	DOWN	DOWN	—	—	Abnormal morphology of the middle ear and ossicles	—	—
Q61191	*Hcfc1*	*HCFC1*	DOWN	UP	—	Sensorineural HI and inner ear functional abnormalities	—	—	—
P38647	*Hspa9*	*HSPA9*	DOWN	DOWN	DFNA42	Aplasia, hypoplasia, and structural abnormalities of the external ear	—	Even‐Plus syndrome MONDO:0014801 OMIM:616854	Microtia
P09535	*Igf2*	*IGF2*	UP	UP	—	Abnormal morphology and positioning of the external ear	—	Beckwith‐Wiedemann syndrome MONDO:0007534 OMIM:130650 Silver‐Russell syndrome 3 MONDO:0014663 OMIM:616489 Silver‐Russell syndrome due to a point mutation ORPHA:397590 Silver‐Russell syndrome due to an imprinting defect of 11p15 MONDO:0016480 ORPHA:231140	Posterior helix pit Low‐set ears Protruding ear
P01872	*Ighm*	*IGHM*	DOWN	—	—	—	—	Autosomal nonsyndromic agammaglobulinemia MONDO:0011096 ORPHA:33110 Agammaglobulinemia 1, autosomal recessive MONDO:0020729 OMIM:601495	Chronic otitis media Abnormal pinna morphology Recurrent otitis media
Q63932	*Map2k2*	*MAP2K2*	UP	—	—	Abnormal morphology and positioning of the external ear	—	Neurofibromatosis‐Noonan syndrome MONDO:0011035 ORPHA:638 Cardiofaciocutaneous syndrome MONDO 15280 ORPHA:1340	Posteriorly rotated ears Abnormal helix morphology Macrotia Posteriorly rotated ears Thickened helices
Q9WTI7	*Myo1c*	*MYO1C*	DOWN	—	—	—	Physiological abnormalities of the auditory, vestibular, and neuronal systems	—	—
Q61753	*Phgdh*	*PHGDH*	DOWN	UP	—	—	—	Neu‐Laxova syndrome 1 MONDO:0009736 OMIM:256520	Low‐set ears Macrotia
Q61207	*Psap*	*PSAP*	UP	DOWN	—	Sensorineural HI and inner ear functional abnormalities	Structural abnormalities of the cochlea, including hair cell and supporting cell malformations Physiological abnormalities of the auditory and vestibular systems, including hair cell dysfunction and impaired otoacoustic responses Neurobiological abnormalities affecting auditory pathways, including altered morphology and innervation of glial cells, Schwann cells, and spiral ganglion neurons (SGNs) Sensorineural HL and vestibular dysfunction	Infantile Krabbe disease MONDO:0016089 ORPHA:206436 Metachromatic leukodystrophy, late infantile form MONDO:0017729 ORPHA:309256 Metachromatic leukodystrophy, adult form MONDO:0017730 ORPHA:309271 Atypical Gaucher disease due to saposin C deficiency MONDO:0012517 ORPHA:309252 Metachromatic leukodystrophy, juvenile form MONDO:0009591 ORPHA:309263	HI Bilateral sensorineural HI Bilateral sensorineural HI Abnormal vestibular function HI Bilateral sensorineural HI
P62267	*Rps23*	*RPS23*	DOWN	—	—	Structural and functional abnormalities of the outer and middle ear (including conductive HI)	—	Brachycephaly, trichomegaly, and developmental delay MONDO:0044311 OMIM:617412	Low‐set ears Overfolded helix Conductive HI
Q9D7A6	*Srp19*	*SRP19*	UP	—	—	—	—	Autosomal dominant severe congenital neutropenia MONDO:0008742 ORPHA:486	Recurrent ear infections
Q9D2G5	*Synj2*	*SYNJ2*	UP	—	—	—	Structural abnormalities of the cochlea, including degeneration of hair cells and spiral ganglion neurons Physiological and electrophysiological abnormalities of the auditory system, including hearing impairment and abnormal auditory brainstem responses Abnormal morphology of hair cells and stereo ciliary bundles Neurobiological abnormalities of the sensory epithelium, including neuronal degeneration		
P21107	*Tpm3*	*TPM3*	UP	DOWN	DFNA49	Abnormal morphology and positioning of the external ear	—	Intermediate nemaline myopathy MONDO:0015736 ORPHA:171433	Low‐set ears

*Note:* This table summarizes 18 proteins prioritized from the proteomic analysis of mouse cochleae following noise overexposure. For each protein, the UniProt accession, mouse and human gene symbols, and direction of regulation in the present MS data set (UP or DOWN) are shown. Regulation in an independent noise‐exposure proteomic study (Jongkamonwiwat et al. 2020) and concordance with the current dataset are indicated. Genomic annotation includes the corresponding human DFN locus when an orthologous deafness locus has been mapped but no causative gene has been identified. Auditory phenotypes were manually curated from human and mouse phenotype databases (HPO and MPO), encompassing structural, functional, and clinical ear‐related features. Associated human disorders and ontology identifiers (MONDO, OMIM, ORPHA) are provided for syndromic conditions linked to each gene.

Abbreviations: HC, hair cells; HI, hearing impairment; SC, supporting cells; SGN, spiral ganglion neurons.

Pharmacological tractability was assessed by annotating the 80‐protein NIHL‐associated signature using the Pharos human pharmacology framework. Two proteins, MAP2K2 and TUBB6, were classified as Tclin, indicating the availability of approved drugs associated with these targets (Supporting Information S2: Table 2G). An additional 13 proteins (AHCY, CES1, CTSD, EIF4E, GARS, GPX4, HSPA9, MAP2, OLA1, PHGDH, PRMT1, SIRT2, and SYNJ2) were classified as Tchem, indicating documented high‐potency chemical ligands according to the Pharos classification. The remaining proteins were classified as Tbio (*n* = 62) or Tdark. Complete pharmacological annotations are provided in Supporting Information S2: Table 2G. These pharmacological annotations indicate the tractability of the corresponding targets but do not imply therapeutic relevance to noise‐induced cochlear damage.

### 3.8 Integrative Functional Classification of Noise‐Responsive Cochlear Proteins

Following the in silico prioritization of DAPs based on genetic, phenotypic, disease, and pharmacological evidence, we next organized the NIHL‐associated proteome into biologically coherent functional frameworks. To this end, we integrated (i) GO and KEGG pathway enrichment analyses (Supporting Information S2: Table 2C); (ii) PPI networks generated using STRING for *Mus musculus* proteins and evaluated in parallel using human orthologs (Supporting Information S2: Table 2H); and (iii) curated functional annotations based on UniProt annotations and the published literature.

To further provide cellular context for the DAP‐encoding genes, we integrated the proteomic classification with the cochlear single‐cell transcriptomic atlas of Jean et al. (2023), examining the representation of Group 1–5 DAP‐encoding genes across defined cochlear and associated cell populations based on their transcriptomic enrichment within each population (Supporting Information S3: Table 3).

This integrative framework organized the 80 DAPs into five major functional groups (Figure 3; Supporting Information S2: Table 2I): (1) Metabolism and Mitochondrial Function; (2) Protein Synthesis and Ribonucleoprotein Complexes; (3) Structural and Synaptic Organization; (4) Cell Polarity and Cytoskeletal Remodeling; and (5) Regulatory, Stress‐Responsive and Specialized Functions. Three proteins (MAP2, PPP1R2, and SIRT2) were retained in both Groups 3 and 4 because their functional annotations supported roles in both structural organization and polarity‐associated cytoskeletal remodeling.

**Figure 3:**
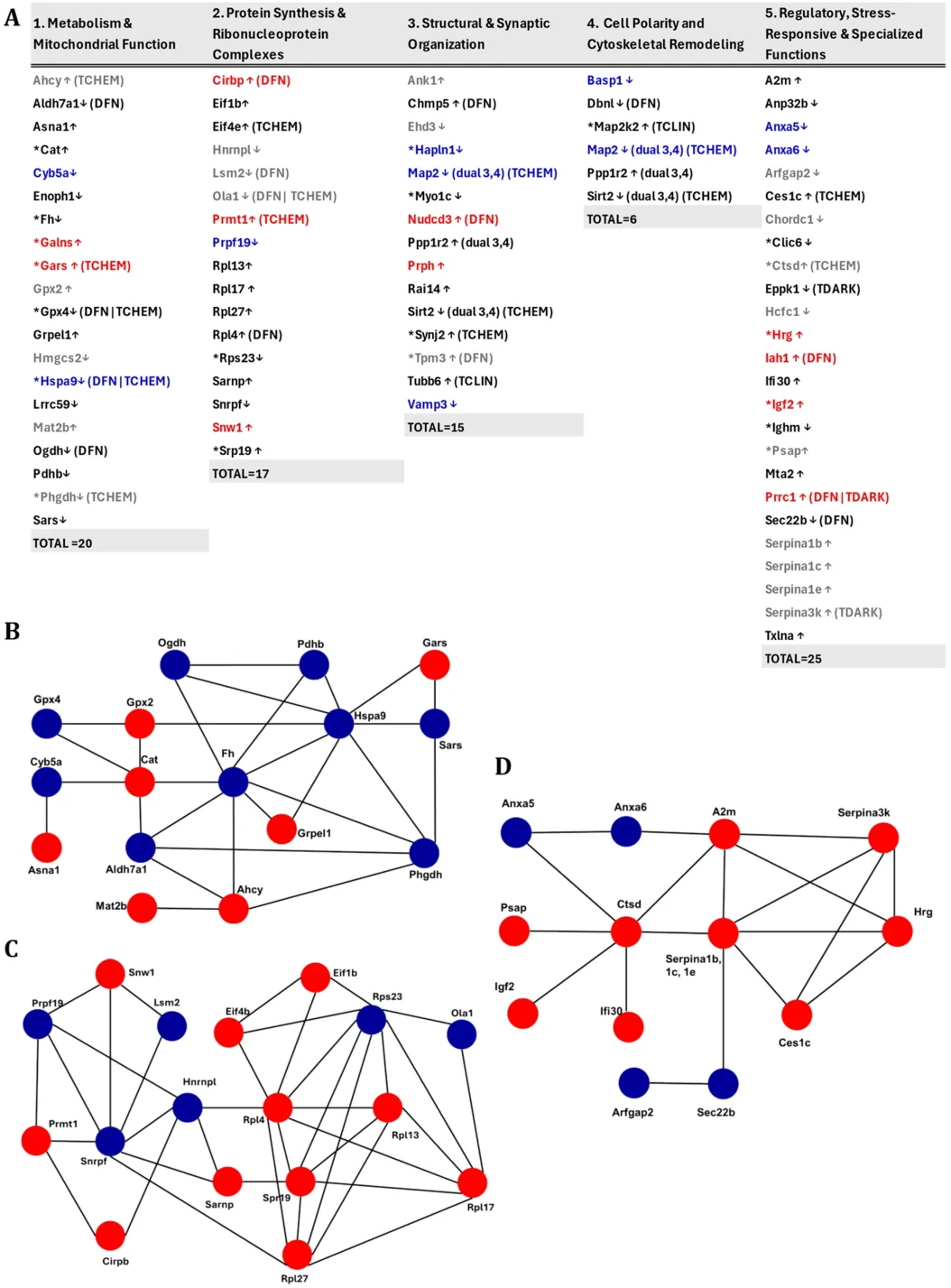
Functional classification and protein–protein interaction networks of DAPs in NIHL. (A) Summary table of the 80 DAPs identified in cochleae from NH and NIHL mice. Proteins are organized into five functional groups based on integrative GO and KEGG enrichment analyses combined with STRING PPI networks. Arrows indicate the direction of regulation observed in the present MS‐based proteomic analysis (↑ upregulated; ↓ downregulated in NIHL). Protein names are color‐coded according to comparison with the cochlear proteomic study by Jongkamonwiwat et al. (2020), which examined protein regulation following high and very‐high noise exposure: black, not identified or validated in the previous study; gray, discordant regulation between studies; red, consistently upregulated; blue, consistently downregulated. Proteins annotated as DFN correspond to those whose human orthologs map to deafness loci without an established causal gene. Drug‐target classification was assigned using the Target Central Resource Database, with TCHEM, TCLIN, and TDARK indicating increasing levels of chemical and clinical target validation; all remaining proteins are classified as TBIO. An asterisk (*) denotes proteins whose genes have been previously associated with hearing alterations or disorders in which HL and/or tinnitus is a reported clinical feature (see Table 1 and Supporting Information S2: Table 2F). (B–D) STRING‐derived protein–protein interaction (PPI) networks illustrating major interconnected modules within selected functional groups: (B) Group 1 – Metabolism and Mitochondrial Function (16 of 20 proteins); (C) Group 2 – Protein Synthesis and Ribonucleoprotein Complexes (17 of 17 proteins); (D) Group 5–Regulatory, Stress‐Responsive, and Specialized Functions (15 of 25 proteins). In each network, nodes represent proteins and edges denote high‐confidence functional associations. Node color reflects regulation in NIHL relative to NH, with red indicating upregulated and blue indicating downregulated proteins.

PPI analysis provided complementary information on the relationships among proteins within the NIHL‐associated signature. The STRING network revealed interactions both within and across the five functional groups, providing an additional layer of functional context for the organization of the NIHL‐associated proteins (Figure 3; Supporting Information S2: Table 2H).

#### 3.8.1 Group 1—Metabolism and Mitochondrial Function

Twenty DAPs were assigned to Group 1 based on functional annotations linking them to metabolic, mitochondrial, and redox‐associated processes (Figure 3A,B; Supporting Information S2: Table 2C,I). Proteins upregulated in NIHL included AHCY, ASNA1, CAT, GALNS, GARS, GPX2, GRPEL1, and MAT2B, whereas ALDH7A1, CYB5A, ENOPH1, FH, GPX4, HMGCS2, HSPA9, LRRC59, OGDH, PDHB, PHGDH, and SARS were downregulated (Supporting Information S2: Table 2A).

The PPI network included 16 of the 20 DAPs in this group (Figure 3B). The interconnected proteins encompassed enzymes involved in mitochondrial energy metabolism, including OGDH, FH, and PDHB; antioxidant and redox‐associated proteins such as CAT, GPX2, GPX4, and CYB5A; and proteins associated with mitochondrial protein homeostasis and metabolic pathways, including HSPA9, GRPEL1, AHCY, MAT2B, ALDH7A1, and PHGDH. Additional proteins within the network included the redox‐regulated ATPase ASNA1 and the aminoacyl‐tRNA synthetases GARS and SARS, illustrating interactions spanning metabolic, redox‐associated, and protein synthesis‐related functions (Figure 3B and Supporting Information S2: Table 2H). Several associations within these functional modules showed high STRING combined scores, including OGDH–PDHB (0.886), FH–OGDH (0.866), and GRPEL1–HSPA9 (0.979). The GARS–SARS pair showed particularly strong support, with a combined score of 0.960 and experimental evidence of 0.869. A smaller redox‐associated cluster involving CAT, GPX2, and GPX4 was also represented, with combined scores ranging from 0.504 to 0.630 (Supporting Information S2: Table 2H).

Four DAPs (GALNS, ENOPH1, HMGCS2, and LRRC59) were not incorporated into the principal PPI network (Figure 3A; Supporting Information S2: Table 2H). Their assignment to Group 1 was instead supported by their functional annotations and by the metabolic processes represented in the GO and KEGG analyses, including lipid and carbohydrate metabolism and mitochondrial‐associated functions (Supporting Information S2: Table 2C).

Single‐cell transcriptomic mapping showed that 14 of the 20 Group 1 DAP‐encoding genes were represented among the top 20% most enriched transcripts in at least one cochlear or associated cell population, with representation across lateral‐wall, supporting‐cell, neuronal, glial, immune, and surrounding‐structure populations (Supporting Information S3: Table 3). This cellular distribution provided context for the metabolic and mitochondrial functions characterizing Group 1, particularly through the representation of several genes in lateral‐wall and supporting‐cell populations.

#### 3.8.2 Group 2—Protein Synthesis and Ribonucleoprotein Complexes

Seventeen DAPs were assigned to Group 2 based on functional annotations related to translation, ribonucleoprotein complex assembly, and amide biosynthesis (Figure 3A; Supporting Information S3: Table 3C,I). Upregulated proteins included ribosomal subunits (RPL4, RPL13, RPL17, RPL27), translation initiation factors (EIF1B, EIF4E), and RNA‑binding or regulatory proteins (CIRBP, SNW1, SARNP, SRP19, PRMT1). Downregulated proteins comprised spliceosomal and RNA‑processing factors (HNRNPL, LSM2, PRPF19, SNRPF), together with the translation‑associated regulator OLA1 and the small ribosomal subunit protein RPS23 (Supporting Information S2: Table 2A).

All 17 Group 2 proteins were incorporated into the PPI network (Figure 3C). One cluster contained ribosomal and translation‑associated proteins, including RPL4, RPL13, RPL17, RPL27, RPS23, EIF1B, and EIF4E. A second cluster consisted of spliceosomal and RNA‑processing factors such as HNRNPL, LSM2, PRPF19, SNRPF, and SNW1, with several interactions supported by high STRING scores (0.994–0.999). Additional RNA‑associated regulators, including CIRBP, SARNP, SRP19, PRMT1, and OLA1, were also present, with PRMT1 connecting to multiple RNA‑processing components (Supporting Information S2: Table 2H).

Cross‑group interactions linked selected Group 2 proteins to metabolic and structural components. For example, PRMT1 interacted with the metabolic enzyme AHCY and the cytoskeletal protein TPM3, bridging RNA‑associated regulatory functions with metabolic and structural pathways represented in the NIHL proteome (Supporting Information S2: Table 2H).

Single‑cell transcriptomic mapping showed that 11 of the 17 Group 2 genes (64.7%) were among the top 20% most enriched transcripts in at least one cochlear or associated cell population, particularly immune cells, supporting cells, lateral‑wall root cells, and glial precursors (Supporting Information S3: Table 3).

#### 3.8.3 Group 3—Structural and Synaptic Organization

Fifteen DAPs were assigned to Group 3 based on enrichment for cellular‑component terms related to supramolecular complexes, supramolecular fibers, and synaptic structures (Figure 3A; Supporting Information S3: Table 2C,I). This group comprises proteins involved in cytoskeletal organization, membrane–cytoskeleton coupling, vesicular trafficking, and synaptic architecture.

Nine proteins were upregulated in NIHL, including cytoskeletal and scaffolding components (ANK1, TPM3, TUBB6, RAI14, PRPH), regulators of endosomal and vesicular trafficking (CHMP5, SYNJ2), the dynein‑associated factor NUDCD3, and the phosphatase inhibitor PPP1R2. Six proteins were downregulated, encompassing elements of endocytic recycling (EHD3, VAMP3), synaptic and extracellular‑matrix organization (HAPLN1), microtubule stability (MAP2), actin‑based motility (MYO1C), and the NAD‑dependent deacetylase SIRT2 (Supporting Information S2: Table 2A).

Unlike the denser intra‑group connectivity observed in Groups 1 and 2, Group 3 displayed limited internal PPI structure, with ANK1–TPM3 representing the only intra‑group interaction (combined score = 0.418). Most connections involving Group 3 proteins occurred across functional groups (Supporting Information S2: Table 2H).

Cross‑group interactions provided additional context for this distributed network. TUBB6 was connected to the mitochondrial chaperone HSPA9 from Group 1 (combined score = 0.519), while SIRT2 showed associations with metabolic proteins from Group 1, including FH (0.569), PHGDH (0.444), and SARS1 (0.592), with experimental evidence of 0.472 for the SIRT2–SARS1 association. TPM3 also interacted with the RNA‐associated regulatory protein PRMT1 from Group 2 (combined score = 0.462). In addition, VAMP3 showed associations with BASP1 from Group 4 (0.447) and SEC. 22B from Group 5, the latter showing a high combined score in mouse (0.884) and experimental evidence of 0.720 (Supporting Information S2: Table 2H).

MAP2, PPP1R2, and SIRT2 were also assigned to Group 4 due to their additional functional associations with polarity and cytoskeletal remodeling (Figure 3A and Supporting Information S2: Table 2I).

Single‑cell transcriptomic mapping showed that 11 of the 15 (73.3%) Group 3 genes were among the top 20% most enriched transcripts in at least one cochlear or associated cell population, with representation across supporting cells (Deiters and pillar cells), lateral‑wall cells, spiral ganglion neurons, and glial populations (Supporting Information S3: Table 3). Structural and synaptic genes such as *Map2, Hapln1, Ank1, Ehd3*, and *Prph* were enriched in spiral ganglion neurons. Cytoskeletal‑ and trafficking‑associated genes, including *Ehd3, Map2, Synj2, Sirt2, Ppp1r2, Rai14*, and *Chmp5*, were enriched in both supporting‑cell and lateral‑wall populations, with *Map2* and *Sirt2* additionally represented in glial populations.

#### 3.8.4 Group 4—Cell Polarity and Cytoskeletal Remodeling

Six DAPs were assigned to Group 4 based on functional annotations related to cell polarity, cytoskeletal organization, and signal‑dependent remodeling (Figure 3A; Supporting Information S2: Table 2C,I). MAP2K2 and PPP1R2 were upregulated in NIHL, whereas BASP1, DBNL, MAP2, and SIRT2 were downregulated (Supporting Information S2: Table 2A). The group includes proteins involved in polarity signaling and cytoskeletal regulation, such as the kinase MAP2K2, the actin‑associated proteins BASP1 and DBNL, and the cytoskeletal regulators MAP2, PPP1R2, and SIRT2.

PPI analysis did not reveal a cohesive intra‑group module among the six proteins. Instead, most interactions involving Group 4 proteins occurred across functional groups (Figure 3A; Supporting Information S2: Table 2H). SIRT2 showed the strongest combined STRING scores with SARS1 (0.592), FH (0.569), and PHGDH (0.444). BASP1 was linked to VAMP3 from Group 3 (0.447), and MAP2 showed an association with SERPINA3 from Group 5 (0.420).

MAP2, PPP1R2, and SIRT2 were additionally assigned to Group 4 based on functional annotations supporting roles in polarity‑associated cytoskeletal remodeling (Figure 3A; Supporting Information S2: Table 2I).

Single‑cell transcriptomic mapping showed that five of the six Group 4 genes (83.3%) were among the top 20% most enriched transcripts in at least one cochlear or associated cell population (Supporting Information S3: Table 3). BASP1, MAP2, SIRT2, and PPP1R2 were enriched in supporting‑cell populations, particularly Deiters and pillar cells, whereas DBNL was enriched among B‑cell transcripts. MAP2 and SIRT2 were also represented in lateral‑wall populations, and BASP1, MAP2, and SIRT2 were represented in glial populations. MAP2K2 was not among the top 20% enriched transcripts in the surveyed populations.

#### 3.8.5 Group 5—Regulatory, Stress‐Responsive and Specialized Functions

Twenty‐five DAPs were assigned to Group 5, representing a heterogeneous set of regulatory, stress‐responsive, membrane‐associated, lysosomal, immune‐associated, and tissue‐ or context‐specific proteins (Figure 3A,D; Supporting Information S3: Table 2C,I). Fifteen proteins were upregulated in NIHL, including the extracellular protease inhibitors A2M, SERPINA1B, SERPINA1C, SERPINA1E, and SERPINA3K; lysosomal and immune‐associated proteins CTSD, IFI30, HRG, IGF2, and PSAP; the metabolic enzymes CES1C and IAH1; and the regulatory proteins MTA2, PRRC1, and TXLNA. Ten proteins were downregulated, including the membrane‐associated proteins ANXA5 and ANXA6, vesicular trafficking proteins ARFGAP2 and SEC. 22B, chromatin‐ and transcription‐associated proteins ANP32B and HCFC1, the cochlear‐ or cell‐type‐associated proteins CLIC6 and EPPK1, the co‐chaperone CHORDC1, and the immune‐associated protein IGHM (Supporting Information S2: Table 2A).

The PPI network included 15 of the 25 Group 5 proteins (Figure 3D). A prominent interconnected module comprised A2M and the SERPINA proteins, with high combined scores observed for SERPINA1B–SERPINA1C (0.956), SERPINA1B–SERPINA1E (0.851), A2M–SERPINA1E (0.803), and A2M–SERPINA1B (0.696). The lysosomal protease CTSD was also connected within this network, showing associations with PSAP (combined score = 0.839), IGF2 (0.591), and ANXA5 (0.414). A second membrane‐ and trafficking‐associated module included ANXA5, ANXA6, ARFGAP2, and SEC. 22B, with the ARFGAP2–SEC. 22B association showing a combined score of 0.722 (Supporting Information S2: Table 2H). Additional cross‐functional associations included CES1C–HRG (combined score = 0.927), indicating connectivity between proteins assigned to the regulatory and stress‐responsive components of the group.

Single‐cell transcriptomic mapping showed that 10 of the 25 Group 5 DAP‐encoding genes (40.0%) were represented among the top 20% most enriched transcripts in at least one cochlear or associated cell population (Supporting Information S3: Table 3). These included *Ighm* in B cells; *Ctsd, Psap*, and *Ifi30* in immune‐associated populations; *A2m, Anxa5*, and *Anxa6* in lateral‐wall populations; and *Clic6, Anxa5*, and *Psap* in supporting‐cell populations*. Igf2, A2m, Clic6, Ctsd, Eppk1, and Anxa5* were also represented in surrounding cochlear structures, while Anxa5 and Ctsd were represented in additional cochlear compartments. Six Group 5 genes (*Ces1c, Hrg, Serpina1b, Serpina1c, Serpina1e*, and *Serpina3k*) were not detected in the P20 atlas (Supporting Information S3: Table 3).

## 4 Discussion

### 4.1 Global Characterization of the Cochlear Proteome Provides a Framework for NIHL Analysis

The cochlea is a highly specialized sensory organ composed of multiple cell populations, many of which are represented by relatively small numbers of cells. Its small size, limited tissue availability, and cellular heterogeneity pose substantial challenges for comprehensive proteomic profiling, particularly when cell‐type‐specific analyses are attempted (Darville and Sokolowski 2013; Darville and Sokolowski 2014; Darville and Sokolowski 2013; Noble et al. 2018). Previous studies have therefore used tissue fractionation, enrichment strategies, or sampling of specific cochlear compartments to increase analytical depth and reduce sample complexity (Darville and Sokolowski 2013; Darville and Sokolowski 2014; Darville and Sokolowski 2013; Jongkamonwiwat et al. 2020).

In the present study, we used whole‐cochlear homogenates to obtain an integrated view of the mature cochlear proteome under NH and noise‐exposed conditions. Because of the limited amount of biological material available from individual cochleae, samples were pooled as four cochleae obtained from two animals for each biological replicate. This approach increased the amount of protein available for MS analysis while maintaining three independent biological replicates per condition, each consisting of pooled cochleae from two animals. Similar pooling strategies have been used in cochlear proteomic studies, reflecting the practical limitations imposed by the small size of the cochlea and the amount of protein that can be recovered from individual samples (Noble et al. 2018; Miao et al. 2021). Pooling, however, also reduces the ability to assess inter‐animal variability and may dilute changes restricted to a small cellular population. Thus, the present dataset should be interpreted as a proteomic profile of pooled cochlear tissue rather than as a cell‐type‐resolved measurement.

Despite these constraints, the whole‐cochlear approach provided an integrated view of the mature mouse cochlear proteome and captured molecular differences between NH and NIHL samples within a largely conserved proteomic background. The large proportion of shared proteins indicates that noise exposure did not substantially alter the overall molecular composition of the cochlea, whereas the separation of the experimental groups by unsupervised clustering supports the presence of condition‐associated differences in protein abundance. Because whole‐cochlear proteomics does not provide cell‐type resolution, signals from more abundant cell populations may predominate, potentially limiting the detection or attribution of changes occurring in smaller or spatially restricted populations. This represents an important consideration when interpreting the present dataset, which provides an integrated view of molecular processes across cochlear compartments rather than a cell‐type‐specific proteomic profile (Jongkamonwiwat et al. 2020; Noble et al. 2018).

Functional classification of the combined NH and NIHL proteome revealed representation of protein classes relevant to cochlear homeostasis. The predominance of metabolic enzymes is consistent with the high energetic demands associated with auditory transduction and maintenance of ion gradients, whereas the representation of cytoskeletal proteins reflects the specialized architecture of hair cells and supporting cells. Protein‐modifying enzymes, molecular chaperones, and scaffold/adaptor proteins further indicate the contribution of protein turnover, proteostasis, and intracellular organization to cochlear function. These features are consistent with the known metabolic demands of the cochlea and its susceptibility to metabolic imbalance and oxidative stress during acoustic injury (Fujimoto and Yamasoba 2014; Jongkamonwiwat et al. 2020; Miao et al. 2022). A substantial fraction of the identified proteins remained unclassified in the PANTHER system, which may reflect incomplete functional annotation or the limited representation of cochlea‐specific biological functions in current annotation resources. This observation also illustrates an important characteristic of global proteomic datasets: although they provide broad molecular coverage, not all detected proteins can be readily assigned to established functional categories.

Functional enrichment analyses revealed substantial convergence between NH and NIHL cochleae in core biological processes, including energy metabolism, cytoskeletal organization, protein synthesis, and complex assembly. This overlap indicates that biological processes fundamental to cochlear function remain broadly represented in both conditions. Redundancy‐reduced GO analyses nevertheless highlighted differences in the relative representation of functional categories, with RNA splicing and cytoskeletal organization emerging as representative terms in the reduced NIHL profile. These differences may reflect remodeling of post‐transcriptional and structural processes in response to acoustic stress, consistent with previous proteomic evidence of cytoskeletal and proteostasis‐related changes following damaging noise exposure (Jongkamonwiwat et al. 2020).

The whole‐cochlear strategy provides an integrated view of molecular changes occurring across the cochlea but does not establish the cellular origin of individual proteins or determine whether observed changes reflect altered protein abundance within a cell type, changes in cellular composition, or both. To provide complementary cellular context, we mapped the identified protein‐coding genes to a published sc‐RNAseq atlas of the mature mouse cochlea (Jean et al. 2023). The similar representation of major cell groups across the NH and NIHL proteomes is compatible with broadly similar representation of these cellular compartments at the tissue level but does not provide direct evidence that cell populations were preserved following noise exposure. Within the limits of bulk proteomics and transcriptomic mapping, these findings are therefore compatible with molecular remodeling occurring across cellular compartments shared between conditions. However, subtle or spatially restricted cell loss, changes affecting small but functionally important populations, and differences in protein detectability cannot be excluded. Importantly, the transcriptomic atlas provides cellular context for the protein‐coding genes detected in the proteome but does not constitute direct evidence of protein localization or cell‐type‐specific regulation.

This interpretation is consistent with previous studies showing that acoustic overexposure induces molecular responses across multiple cochlear cell populations and biological processes. Quantitative proteomic analyses have identified cytoskeletal and proteostasis‐related changes following damaging noise exposure, together with increased abundance of protein synthesis machinery during recovery (Jongkamonwiwat et al. 2020). Complementary metabolomics and molecular studies have implicated metabolic dysfunction, oxidative stress, and autophagy‐related responses in NIHL (Miao et al. 2021). More recently, time‐series transcriptomic analyses have demonstrated dynamic activation of immune and tissue‐remodeling programs during NIHL progression and recovery (Sels et al. 2025).

Among the identified proteins, 25 corresponded to orthologs of genes previously implicated in human hereditary HL (Shearer and Smith 2012; Walls et al. 2025). Their detection in an unbiased, whole‐cochlear dataset indicates that proteins encoded by genes implicated in human hereditary hearing loss can be captured without targeted enrichment. Their representation across diverse functional categories and transcriptomically defined cochlear cell populations is consistent with a systems‐level view of hearing loss involving interconnected cellular processes rather than isolated molecular pathways (Fujimoto and Yamasoba 2014; Ingham et al. 2019; Kalra et al. 2020; Tan and Song 2023).

Taken together, these findings show that whole‐cochlear proteomics can provide a broad overview of the mature cochlear proteome, capturing core biological functions as well as proteins encoded by genes associated with human hearing phenotypes. The comparison between NH and NIHL samples further revealed condition‐associated molecular differences within this broadly conserved proteomic background, providing a basis for subsequent analysis of the protein changes and functional pathways associated with the NIHL response.

### 4.2 DAPs Reveal Coordinated Molecular Remodeling in NIHL

By integrating quantitative proteomic changes with functional enrichment, PPI analysis, comparison with an independent cochlear proteomic data set, cochlear single‐cell transcriptomic context, and human genetic annotation, we define a coherent NIHL‐associated proteomic landscape comprising 80 DAPs organized into five major functional modules. Nearly half of these DAPs (39 of 80; 48.8%) were also identified as differentially regulated in an independent noise‐exposure dataset (Jongkamonwiwat et al. 2020), providing external support for a subset of the identified protein changes. Concordant and divergent regulation patterns likely reflect differences in exposure intensity and experimental context, underscoring NIHL as a dynamic process rather than a binary molecular state. Together, these findings indicate that the NIHL‐associated proteome encompasses interconnected metabolic, translational, structural, signaling, and stress‐responsive processes rather than a single dominant molecular pathway (Fetoni et al. 2019; Jongkamonwiwat et al. 2020; Tan and Song 2023).

The functional modules described below were defined by the convergence of differential abundance, functional enrichment, PPI connectivity, and biological context rather than by enrichment analysis alone. This organization provides a framework for interpreting the 80 DAPs at the level of interconnected processes, while recognizing that the resulting modules are biologically informed and hypothesis‐generating rather than evidence of causal pathways. Next, each functional module was examined in detail, beginning with metabolic and mitochondrial remodeling.

### 4.3 Metabolic and Mitochondrial Remodeling in NIHL

Group 1 comprises 20 DAPs associated with central metabolism, mitochondrial function, and redox homeostasis. Except for ENOPH1, GALNS, HMGCS2, and LRRC59, these proteins formed a highly interconnected PPI network, indicating extensive functional connectivity among mitochondrial enzymes, redox regulators, and biosynthetic pathways. Up‐ and downregulated proteins were interspersed throughout the network, suggesting that the metabolic response was not characterized by uniform pathway suppression but rather by selective perturbation of individual metabolic nodes (Figure 3B; Supporting Information S2: Table 2H). Consistent with this interpretation, comparison with the independent cochlear noise‐response proteome of Jongkamonwiwat et al. (2020) identified nine Group 1 proteins that were also differentially regulated, including five with concordant regulation (CYB5A, GALNS, GARS, HSPA9, MAT2B) and four with discordant regulation (AHCY, GPX2, HMGCS2, PHGDH). Thus, the external dataset provides independent proteomic support for selected components of the module, whereas the discordant responses indicate that individual metabolic proteins may vary according to experimental context.

Mitochondrial dysfunction is a well‐established feature of cochlear pathology following acoustic trauma (Fetoni et al. 2019; Jongkamonwiwat et al. 2020; Miao et al. 2022; Li et al. 2023; Tan and Song 2023). In this context, downregulation of the HSPA9 protein, a mitochondrial chaperone involved in protein import and folding, consistent with altered mitochondrial proteostasis and may indicate increased cellular vulnerability to metabolic stress (Texier et al. 2023). The concordant reduction of HSPA9 in the independent cochlear proteomic dataset further supports the reproducibility of this alteration (Jongkamonwiwat et al. 2020; Supporting Information S2: Table 2B). Although the functional significance of altered HSPA9 abundance in the cochlea has not been experimentally established, its established roles in mitochondrial protein homeostasis and cellular stress provide relevant biological context (Texier et al. 2023). The human *HSPA9* gene is located within the DFNA42 interval but outside the mapped DFNA54 and DFNB60 intervals (Walls et al. 2025; Supporting Information S2: Table 2D), and biallelic pathogenic variants in *HSPA9* cause EVEN PLUS syndrome (MONDO#0014801, OMIM#616854) (Table 1 and Supporting Information S2: Table 2F). Together, the observed reduction of HSPA9, its concordant regulation in an independent cochlear proteomic dataset, and its established role in mitochondrial protein homeostasis identify HSPA9 as a biologically plausible candidate for further investigation in the cochlear response to acoustic stress.

Further evidence of altered mitochondrial and intermediary metabolism was provided by downregulation of FH and OGDH, together with PDHB and HMGCS2. FH and OGDH are directly involved in the tricarboxylic acid cycle, whereas PDHB links glycolytic carbon metabolism to the TCA cycle and HMGCS2 participates in mitochondrial ketogenesis. Pathogenic variants in the corresponding human genes cause inherited metabolic disorders and, for some of these genes, are associated with ear or hearing‐related phenotypes (Supporting Information S2: Table 2E,F). Rather than indicating complete metabolic failure, their coordinated reduction within the broader network is consistent with altered mitochondrial and intermediary metabolic capacity following acoustic stress. OGDH is also remarkable because the corresponding human gene was positioned within a mapped deafness locus lacking a definitive causal gene (Supporting Information S2: Table 2D; Walls et al. 2025), providing additional genetic context for its reduced abundance in the present proteome. Notably, HMGCS2 showed discordant regulation between the present and independent datasets (Jongkamonwiwat et al. 2020, Supporting Information S2: Table 2B), suggesting that individual components of the metabolic response may vary according to the experimental context rather than exhibiting uniform directional regulation.

An alteration in antioxidant defenses was also evident within this module. GPX4, a key regulator of lipid peroxide detoxification and ferroptosis suppression, was downregulated in NIHL. The corresponding human *GPX4* gene was considered in the positional analysis in relation to the DFNB72 interval, although it lies outside the mapped interval (Walls et al. 2025). Thus, the positional relationship should be interpreted as genetic context rather than evidence that GPX4 is the causal gene for DFNB72. Its biological relevance is nevertheless supported by experimental evidence implicating GPX4‐dependent ferroptotic mechanisms in cochlear injury, including hair‐cell and synaptic dysfunction in ototoxic models (Huang et al. 2024; Liu et al. 2024; Chen et al. 2025). However, these studies do not establish that the reduction in GPX4 observed here reflects activation of ferroptotic mechanisms in the noise‐exposed cochlea.

In contrast, CAT was upregulated (Supporting Information S2: Table 2A), which may reflect an adaptive antioxidant response to increased oxidative burden. This interpretation is compatible with evidence linking catalase‐related genetic variation to susceptibility to NIHL and with experimental evidence supporting the importance of antioxidant defenses in limiting cochlear injury (Youn et al. 2017; Wu et al. 2023). The opposing regulation of GPX4 and CAT is therefore consistent with altered redox homeostasis, although increased CAT abundance alone does not establish effective antioxidant compensation, increased lipid peroxidation, or ferroptotic cell death. More broadly, these findings are consistent with the established contribution of oxidative stress and impaired antioxidant defenses to cochlear vulnerability following acoustic and other toxic insults (Tan and Song 2023; Li et al. 2023; Wu et al. 2023).

Disruption of one‐carbon and amino‐acid metabolism further characterized this metabolic network. AHCY, MAT2B, and PHGDH, enzymes involved in methyl‐group and serine‐associated metabolic pathways, displayed context‐dependent regulation across datasets, suggesting dynamic pathway remodeling under acoustic stress (Bao et al. 2016; Ducker and Rabinowitz 2017). Specifically, MAT2B showed concordant upregulation, whereas AHCY and PHGDH displayed discordant regulation between the two proteomic datasets (Jongkamonwiwat et al. 2020, Supporting Information S2: Table 2). This pattern suggests that individual components of interconnected metabolic pathways may respond differently according to the experimental context rather than exhibiting uniform pathway‐level regulation. Although NIHL is an acquired condition, the involvement of related metabolic pathways in developmental and congenital disorders provides additional biological context for their potential relevance to auditory homeostasis. In particular, PHGDH deficiency is associated with congenital developmental abnormalities that include external ear abnormalities, supporting a broader connection between serine metabolism and ear development (Shaheen et al. 2014; Malesci et al. 2023).

The downregulation of ALDH7A1 is also notable in the context of metabolic and oxidative‐stress remodeling. The corresponding human *ALDH7A1* gene lies within the mapped DFNB60 interval but outside the DFNA54 interval (Supporting Information S2: Table 2D). ALDH7A1 contributes to the detoxification of lipid peroxidation‐derived aldehydes and to cellular resilience against oxidative stress (Brocker et al. 2011). In the cochlea, where oxidative stress is an important component of hair cell and neuronal injury, reduced ALDH7A1 abundance therefore provides a biologically plausible target for further investigation, although its contribution to cochlear stress responses remains to be established (Teraoka et al. 2024).

Similarly, GALNS showed concordant upregulation in the independent cochlear proteomic dataset (Jongkamonwiwat et al. 2020, Supporting Information S2: Table 2B), linking lysosomal glycosaminoglycan catabolism and extracellular matrix homeostasis to auditory function. Although GALNS was not incorporated into the principal PPI network, its assignment to Group 1 was supported by GO and KEGG enrichment. *GALNS* deficiency causes mucopolysaccharidosis IVA (MONDO#0009659, OMIM#253000), a disorder characterized by glycosaminoglycan accumulation and skeletal and connective‐tissue abnormalities in which hearing impairment is well documented (Clarke et al. 2016; Yi et al. 2022; Hendriksz et al. 2013; Wolfberg et al. 2020). Thus, the concordant GALNS increase provides reproducible protein‐level evidence of its alteration following noise exposure, while the human disease literature provides independent auditory context that supports the biological relevance of this finding.

The concordant upregulation of GARS in the present and independent cochlear proteomic data sets provides additional support for the reproducibility of metabolic changes following noise exposure (Jongkamonwiwat et al. 2020; Supporting Information S2: Table 2B). GARS encodes glycyl‐tRNA synthetase, an aminoacyl‐tRNA synthetase that functions in both cytosolic and mitochondrial protein synthesis, thereby linking aminoacylation with cellular and mitochondrial protein homeostasis (Boczonadi et al. 2018). Pathogenic *GARS* variants have primarily been associated with neuronal and neuromuscular phenotypes, and experimental studies have linked *GARS* dysfunction to altered mitochondrial function and neuronal vulnerability (Seburn et al. 2006; Boczonadi et al. 2018). The concordant increase of GARS across the two cochlear proteomic data sets adds reproducible evidence of altered protein homeostasis within the broader metabolic and mitochondrial proteomic response to acoustic stress.

The single‐cell transcriptomic atlas further provided a broad cellular context for these metabolic genes, with representation across lateral‐wall, supporting‐cell, neuronal, glial, immune, and surrounding cochlear populations (Supporting Information S3: Table 3; Jean et al. 2023). These data indicate that the genes corresponding to the identified proteins are transcriptionally active across multiple cochlear compartments under basal conditions, but they do not establish the cellular origin of the protein changes or demonstrate cell‐type‐specific responses to noise exposure.

From a translational perspective, this group contains several pharmacologically tractable proteins classified as TCHEM, including the proteins AHCY, GARS, GPX4, HSPA9, and PHGDH (Supporting Information S2: Table 2G). Although these annotations indicate different levels of pharmacological tractability, their therapeutic relevance to HL remains to be established. Their convergence within the noise‐responsive metabolic network identifies potential candidates for future mechanistic studies of mitochondrial quality control, redox balance, and biosynthetic capacity in the cochlea.

Overall, Group 1 defines a highly interconnected metabolic and mitochondrial module in which energetic metabolism, redox homeostasis, and protein quality control converge. The combination of differential abundance, network connectivity, human genetic context, and independent proteomic evidence supports these processes as prominent components of the molecular response to noise exposure, while the specific contribution of individual proteins to cochlear injury remains to be determined.

### 4.4 Translational Control and RNA Processing Are Remodeled in the Noise‐Exposed Cochlea

Group 2 comprises 17 DAPs involved in translation initiation, ribonucleoprotein complex assembly, and RNA processing. All proteins in this group were incorporated into a single PPI network, revealing extensive network connectivity among the proteins comprising the module (Figure 3C; Supporting Information S2: Table 2H). Functional enrichment further supported this organization, with Group 2 proteins enriched in translation, amide metabolic and biosynthetic processes, peptide biosynthesis, and ribonucleoprotein‐related functions (Supporting Information S2: Table 2C,I).

A defining feature of this group is its opposing regulation. Ribosomal proteins (RPL4, RPL13, RPL17, RPL27), translation initiation factors (EIF1B, EIF4E), and selected RNA‐binding or regulatory proteins (CIRBP, SNW1, SARNP, SRP19, PRMT1) were predominantly upregulated. In contrast, core components of spliceosomal and RNA‐processing complexes (HNRNPL, LSM2, PRPF19, SNRPF), together with the translation‐associated proteins OLA1 and RPS23, were downregulated (Supporting Information S2: Table 2A). This opposing regulation within a single network is more consistent with selective remodeling of translational and posttranscriptional processes than with a generalized increase in translation or uniform suppression of RNA processing (Figure 3C; Supporting Information S2: Table 2H). Such selective remodeling of translational and RNA‐processing components is compatible with established stress responses, in which protein synthesis and RNA metabolism are reprogrammed according to cellular conditions (Spriggs et al. 2010; Zhu et al. 2016). Since translation rates, ribosome activity, and translational flux were not assessed, it is not possible to conclude whether global protein synthesis increased or remained unchanged.

The upregulation of EIF4E, a central cap‐binding component of the translation‐initiation machinery, identifies a potentially relevant regulatory component within this module. In the human pharmacological annotation, EIF4E was classified as Tchem (Supporting Information S2: Table 2G), indicating documented chemical tractability of the corresponding target. Experimental studies in pain‐related models have shown that modulation of the MNK–eIF4E axis can influence injury‐induced neuronal plasticity and nociceptive sensitization (Moy et al. 2017). However, these studies did not examine acoustic trauma or NIHL, and no direct evidence currently establishes that modulation of EIF4E signaling modifies cochlear injury or HL after noise exposure. Thus, the Tchem classification of EIF4E provides pharmacological context for future mechanistic studies of translational regulation in acoustic injury.

CIRBP was upregulated in the present study and showed concordant regulation in the independent cochlear proteomic data set (Jongkamonwiwat et al. 2020; Supporting Information S2: Table 2A,B), providing independent support for the reproducibility of its differential abundance within this stress‐responsive signature. As a conserved stress‐responsive RNA‐binding protein involved in mRNA stability and translation (Corre and Lebreton 2024), this concordant protein‐level change provides biological context for the post‐transcriptional remodeling observed in the noise‐exposed cochlea. The opposing regulation of spliceosomal components such as LSM2, SNRPF, and PRPF19 further supports remodeling of RNA‐processing machinery within the module. PRPF19 showed concordant downregulation in the independent cochlear proteomic data set, whereas SNW1 was concordantly upregulated and displayed high‐confidence associations with PRPF19 and SNRPF within the Group 2 network (Figure 3C; Supporting Information S2: Table 2B,H). Together, these observations provide independent support for changes affecting both translational and RNA‐processing components of the module, without implying a uniform change in global splicing activity.

Within this regulatory landscape, OLA1 emerged as a context‐dependent component of the module. OLA1 is a conserved ribosome‐associated ATPase involved in translation regulation and cellular stress responses (Landwehr et al. 2021; Yu et al. 2025). It was downregulated in the present study but showed discordant regulation in the independent cochlear proteomic data set (Jongkamonwiwat et al. 2020; Supporting Information S2: Table 2B). Although its established role in translational regulation provides biological context for its position within Group 2, its divergent regulation across data sets highlights the context‐dependent behavior of this component of the translational response.

The interaction network revealed two associated components, one centered on ribosomal proteins and translation‐initiation factors and the other on spliceosomal and RNA‐processing proteins, with regulatory proteins such as SNW1 and PRMT1 connecting these components within the network (Figure 3C; Supporting Information S2: Table 2H). SNW1 displayed interactions with PRPF19 and SNRPF within our network, consistent with the established role of SNW1 as a spliceosomal component and transcriptional co‐regulator (Olgun and Muyan 2018; Verma et al. 2019). Interactions with MAT2B, AHCY, PHGDH, and OGDH from Group 1 further connected RNA‐and protein‐synthesis‐associated processes with the metabolic remodeling described above, illustrating connectivity between proteins assigned to the two functional groups. These cross‐group interactions place translational and RNA‐processing proteins within the broader metabolic network represented in the NIHL‐associated proteome (Liu and Qian 2014; Kitama et al. 2025).

PRMT1 emerged as a prominent regulatory component within this network. Its identification was supported by multiple complementary observations, including increased abundance in the present cochlear proteome, concordant upregulation in the independent cochlear proteome, interactions with multiple RNA‐processing proteins, and representation of the corresponding transcript across cochlear cell populations in the single‐cell atlas (Supporting Information S2: Table 2A,B,H and Supporting Information S2: Table 3; Figure 3C). PRMT1 is a major type I protein arginine methyltransferase with established roles in RNA‐associated regulation, translation, and cellular stress‐responsive pathways (Tang et al. 2000; Thiebaut et al. 2021). Together, these convergent observations place PRMT1 at the interface of translational and RNA‐processing responses represented in the noise‐exposed cochlear proteome. PRMT1 was also classified as Tchem in the human pharmacological annotation (Supporting Information S2: Table 2G), indicating documented chemical tractability of this target and providing an additional resource for future mechanistic studies. Within the PPI network, PRMT1 interacted with RNA‐processing components and with the metabolic enzyme AHCY, while its interaction with the cytoskeletal protein TPM3 extended its connectivity beyond Group 2 (Supporting Information S2: Table 2H; Figure 3C). These network associations further position PRMT1 at the intersection of RNA‐processing, translational, metabolic, and cytoskeletal processes, consistent with broader evidence linking PRMT‐regulated networks to mRNA splicing and translation (Wei et al. 2021).

Comparison with the independent cochlear proteomic dataset of Jongkamonwiwat et al. (2020) identified seven Group 2 proteins that were also differentially regulated (Supporting Information S2: Table 2B). Four proteins (CIRBP, PRMT1, SNW1, PRPF19) showed concordant regulation across data sets, whereas three (HNRNPL, LSM2, OLA1) showed discordant regulation. The four concordant proteins span both major functional components of the module: CIRBP, PRMT1, and SNW1 were upregulated, whereas PRPF19 was downregulated. This cross‐study agreement supports reproducibility of selected elements of the translational and RNA‐processing signature rather than uniform replication of the entire module. The discordant regulation of HNRNPL, LSM2, and OLA1 indicates that not all components of the Group 2 signature were consistently altered across studies. Differences in noise exposure paradigms, sampling time points, age, and sequencing strategies may contribute to such differences (Sels et al. 2025).

Several Group 2 proteins also corresponded to human orthologs considered in the positional analysis of unresolved HL loci (Supporting Information S2: Table 2D). The human *CIRBP* gene lies outside the mapped DFNB72 interval, whereas *OLA1* lies within the mapped DFNB27 interval. *LSM2* lies outside the mapped DFNA21 and DFNA31 intervals but within the mapped *OTSC3* interval, while *RPL4* lies outside the mapped USH1H interval but relatively close to its mapped boundary (Walls et al. 2025; Supporting Information S2: Table 2D). These positional relationships provide additional genetic context for selected Group 2 proteins, but they do not establish causality for the corresponding HL loci. Because unresolved intervals may contain multiple candidate genes, positional proximity alone cannot establish functional involvement. These positional relationships therefore provide a framework for prioritizing genes for further genetic and functional investigation.

RPS23 provides relevant biological links between translational regulation and auditory dysfunction. The protein was downregulated in the present NIHL proteome and belongs to the small ribosomal subunit (Supporting Information S2: Table 2A). In humans, pathogenic *RPS23* variants have been associated with a ribosomopathy characterized by HL and impaired translational fidelity, with mutant cells showing increased sensitivity to oxidative stress (Paolini et al. 2017; Table 1 and Supporting Information S2: Table 2F). These findings provide independent biological context for the potential relevance of translational fidelity to auditory vulnerability.

Single‐cell transcriptomic data showed that Group 2 genes were represented across multiple cochlear cell populations (Supporting Information S3: Table 3; Jean et al. 2023). This broad distribution is consistent with the widespread cellular requirement for translational and RNA‐processing machinery. Because the atlas represents the basal transcriptomic state of normal‐hearing P20 cochleae, it provides cellular context for the proteins identified in the present proteome but does not establish their cellular origin or noise‐induced transcriptional regulation. Taken together with the proteomic, network, and cross‐study evidence, these data support the interpretation of Group 2 as a distributed remodeling of translational and RNA‐processing machinery in the noise‐exposed cochlea.

Overall, Group 2 is characterized by integrated but non‐uniform changes across translational and RNA‐processing machinery. The reproducibility of selected proteins across an independent cochlear proteomic dataset, together with their network connectivity and biological context, supports Group 2 as a proteomic signature of selective remodeling of translational and RNA‐processing machinery in the noise‐exposed cochlea.

### 4.5 Structural and Synaptic Remodeling in the Noise‐Exposed Cochlea

Group 3 comprises 15 DAPs associated with cytoskeletal organization, synaptic structures, membrane‐cytoskeleton coupling, and intracellular trafficking. Functional enrichment identified cellular‑component terms related to supramolecular complexes, supramolecular fibers, and synaptic structures, consistent with functions related to cytoskeletal organization, membrane–cytoskeleton coupling, vesicular trafficking, and synaptic maintenance (Figure 3A; Supporting Information S2: Table 2C,I). These processes are central to the maintenance of hair‐cell architecture, synaptic organization, and intracellular transport within the auditory sensory epithelium and therefore provide a functional framework for interpreting the Group 3 signature (Hickman et al. 2020; Mohamad et al. 2024; Wu et al. 2024; Table 1).

Six proteins were downregulated (EHD3, HAPLN1, MAP2, MYO1C, SIRT2, and VAMP3), whereas nine were upregulated in NIHL (ANK1, CHMP5, NUDCD3, PRPH, PPP1R2, RAI14, SYNJ2, TPM3, and TUBB6) (Supporting Information S2: Table 2A). This mixed regulatory pattern suggests selective remodeling of structural and trafficking pathways rather than a uniform impairment of cytoskeletal machinery. Group 3 exhibited limited internal PPI connectivity, with ANK1 and TPM3 forming the main intragroup interaction, while several proteins engaged in cross‑group associations (Supporting Information S2: Table 2H). Notably, TPM3 and TUBB6 linked Group 3 to metabolic and mitochondrial‑associated proteins from Group 1; VAMP3 connected structural processes with regulatory and membrane‑trafficking proteins from Groups 4 and 5; and TPM3 interacted with PRMT1 from Group 2. These cross‑group relationships support the interpretation of Group 3 as a distributed structural interface integrated with metabolic and regulatory pathways rather than a discrete molecular complex.

Comparison with the independent cochlear proteomic dataset of Jongkamonwiwat et al. (2020) identified eight Group 3 proteins that were also differentially regulated. HAPLN1, MAP2, and VAMP3 showed concordant downregulation, whereas PRPH and NUDCD3 were concordantly upregulated; ANK1, TPM3, and EHD3 showed discordant regulation (Supporting Information S2: Table 2B). Notably, the concordant proteins included components of extracellular matrix organization, cytoskeletal structure, vesicular trafficking, and neuronal integrity, indicating that the overlap extended across distinct aspects of the Group 3 signature.

HAPLN1 is a link protein involved in the organization of hyaluronan–proteoglycan complexes and specialized extracellular matrix structures, including perineuronal and perisynaptic matrices (Oohashi et al. 2015; Kwok et al. 2010). In the cochlea, HAPLN1 is enriched in extracellular matrix baskets surrounding the basolateral poles of inner hair cells, where these structures are associated with synaptic contacts (Sonntag et al. 2018). In a rat model of NHIL Kim et al. (2025) reported a tendency toward reduced HAPLN1 following noise exposure, providing independent evidence that this extracellular matrix component is altered following acoustic trauma. Consistent with this observation, HAPLN1 was downregulated in our noise‐exposed cochleae (Supporting Information S2: Table 2A). The concordant reduction across independent proteomic data sets, together with its localization to IHC‐associated extracellular matrix structures, links HAPLN1 to extracellular matrix remodeling at cochlear synaptic regions. In the cochlear single‐cell atlas of Jean et al. (2023), *Hapln1* was among the highly expressed transcripts in spiral ganglion neuron populations (Supporting Information S3: Table 3), providing additional cellular context for its detection in the whole‐cochlea proteome. Although the functional consequences of reduced HAPLN1 abundance for cochlear synaptic integrity remain to be established, these findings identify extracellular matrix remodeling as a component of the structural changes represented in Group 3.

SYNJ2 provides a stronger direct auditory context. Two independent mouse models have demonstrated that *Synj2* disruption causes progressive HL accompanied by stereocilia abnormalities and HCs degeneration (Manji et al. 2011; Martelletti et al. 2020). Human cochlear studies have detected *SYNJ2* in spiral ganglion neurons and outer spiral bundles, while human genetic studies have associated *SYNJ2* with age‐related HL, including rare‐variant associations (Liu et al. 2021; Cornejo‐Sanchez et al. 2023). These findings support SYNJ2 as a component of cochlear auditory biology, although current human evidence represents association rather than established Mendelian HL causality. The increased abundance of SYNJ2 in the present NIHL proteome therefore adds a protein‐level change to an existing body of auditory evidence (Table 1). Notably, *Synj2*‐deficient mice did not show detectable defects in inner‐hair‐cell exocytosis or endocytosis, indicating that the present increase should not be interpreted specifically as evidence of altered ribbon‐synapse vesicle trafficking (Martelletti et al. 2020).

The reduction of MYO1C provides an additional connection between Group 3 and hair‐cell mechanotransduction. MYO1C is expressed in cochlear hair cells and localizes to stereocilia, where it contributes to the molecular machinery underlying slow adaptation of mechanoelectrical transduction (Dumont et al. 2002; Batters et al. 2004; Phillips et al. 2006). These established functions provide biological context for the reduced MYO1C abundance observed here in relation to cytoskeletal and mechanotransduction remodeling following acoustic stress. However, the human gene–disease relationship remains unresolved. Variants in MYO1C were initially reported in individuals with hearing loss, and subsequent functional studies showed that some of these variants, including p.R156W, alter MYO1C motor properties (Zadro et al. 2009; Adamek et al. 2010). Nevertheless, the MYO1C‐HL association was classified as Disputed by the ClinGen Hearing Loss Clinical Domain Working Group, and a recent review likewise considers this gene–disease relationship unresolved (DiStefano et al. 2019; Miyoshi et al. 2024). The present finding therefore supports MYO1C as a biologically plausible component of cochlear mechanotransduction and structural remodeling, rather than as an established causal gene for NIHL.

MAP2 provides complementary evidence for cytoskeletal involvement in cochlear function. MAP2 is expressed in mouse cochlear hair cells and is involved in microtubule‐associated processes in the auditory system (Ladrech and Lenoir 2002). Recent experimental evidence showed that *Map2* deficiency causes progressive high‐frequency HL in mice, accompanied by reduced numbers of basal outer hair cells; MAP2 was detected in both inner and outer hair cells in the wild‐type mouse cochlea (Shin'ya et al. 2025). Earlier studies also showed dynamic changes in MAP2 expression and localization following cochlear ototoxic or excitotoxic injury, including changes in hair cells after amikacin exposure and in auditory neurons during excitotoxic repair in rodents (Ladrech and Lenoir 2002; Ladrech et al. 2003). The concordant reduction of MAP2 in the present and independent proteomic data sets further supports the reproducibility of this protein‐level change in noise‐exposed mouse cochleae (Supporting Information S2: Table 2B). In the Jean et al. atlas, *Map2* transcripts were detected across supporting cell and spiral ganglion neuron populations in the mouse cochlea (Supporting Information S3: Table 3), providing additional cellular context for the detected protein. Taken together, the reduction of MAP2 in the present and independent proteomic datasets, together with its cochlear expression and the hearing phenotype observed following *Map2* deficiency in mice, supports MAP2 as a biologically relevant component of cochlear cytoskeletal organization.

The reduction of EHD3 and VAMP3 further implicates membrane recycling and vesicular turnover within the Group 3 signature. These processes are relevant to synaptic maintenance and have been implicated in cochlear synaptopathy, in which synaptic loss may occur after noise exposure without extensive hair‐cell loss (Liberman and Kujawa 2017). The concordant reduction of VAMP3 across independent datasets strengthens its consideration as a reproducible component of the structural signature, whereas the discordant behavior of EHD3 suggests greater context dependence (Supporting Information S2: Table 2B). Together, these changes are compatible with remodeling of vesicular and membrane‐associated maintenance processes within the noise‐exposed cochlea.

Several other Group 3 proteins were increased, including TPM3, TUBB6, and NUDCD3. Their combined regulation is more conservatively interpreted as remodeling of cytoskeletal and intracellular transport machinery than as evidence of a uniform adaptive response. NUDCD3 is associated with dynein‐dependent intracellular transport, whereas TPM3 and TUBB6 participate in actin‐ and microtubule‐associated structural organization. Three Group 3 proteins also correspond to human orthologs positioned relative to unresolved hereditary HL loci: *NUDCD3* lies within the mapped DFNB44 interval, whereas *CHMP5* and *TPM3* are positioned outside the mapped *OTSC8* and DFNA49 intervals, respectively (Supporting Information S2: Table 2D). These positional relationships provide additional genetic context for the observed protein changes and may help prioritize genes for further investigation.

TPM3 warrants additional consideration because it combines cytoskeletal function with human disease context. The human *TPM3* gene is associated with intermediate nemaline myopathy (MONDO#0015736; ORPHA#171433), a disorder in which craniofacial abnormalities including low‐set ears have been reported (Supporting Information S2: Table 2D; Table 1). These phenotypic annotations provide biological context for the altered abundance of a cytoskeletal protein involved in actin organization, but they do not constitute evidence that *TPM3* is a HL gene or that it mediates NIHL. This distinction is particularly important because the human ortholog lies outside the mapped DFNA49 interval. Thus, *TPM3* is best regarded here as a cytoskeletal candidate with relevant human phenotypic context rather than as a causal auditory gene.

PRPH provides a more direct link between neuronal cytoskeletal organization and susceptibility to acoustic injury. Peripherin is a type III intermediate filament protein expressed in a subset of spiral ganglion neurons in the adult mouse cochlea (Lee et al. 2025), where it contributes to neuronal structural organization (Millecamps et al. 2006). Experimental studies in *Prph*‐deficient mice demonstrated increased vulnerability to NIHL, supporting a functional relationship between peripherin‐dependent neuronal organization and susceptibility to acoustic injury (Cederholm et al. 2022). The concordant increase of PRPH in the independent proteomic data set further supports its reproducibility as a component of the neuronal structural response (Supporting Information S2: Table 2B).

Several Group 3 proteins have also been implicated in neuronal and neurodegenerative processes, providing a broader biological context for the structural signature. ANK1 dysregulation has been associated with Alzheimer's disease neuropathology (De Jager et al. 2014; Lunnon et al. 2014), EHD3 participates in vesicular pathways linked to amyloid processing (Buggia‐Prévot et al. 2013), and MAP2 is involved in microtubule organization and dendritic stability in neuronal systems (Pîrşcoveanu et al. 2017). SIRT2 likewise has context‐dependent roles in oxidative stress, neuroinflammation, and neurodegenerative processes (Sola‐Sevilla & Puerta 2024; Sola‐Sevilla et al. 2025). Beyond these individual protein associations, oxidative stress, mitochondrial dysfunction, neuroinflammation, and synaptic injury have been implicated in both noise‐induced cochlear injury and neurodegenerative disease (Paciello et al. 2023; Tan and Song 2023). In an experimental Alzheimer's disease model, noise‐induced hearing loss was additionally associated with increased tau phosphorylation, neuroinflammation, redox imbalance, and earlier cognitive impairment, providing experimental evidence that noise‐related auditory injury can influence pathways relevant to neurodegenerative pathology (Paciello et al. 2021).

The cellular distribution of Group 3 genes in the cochlear single‐cell atlas provides additional cellular context for the corresponding proteins across distinct cochlear compartments. SYNJ2, MAP2, SIRT2, PPP1R2, and RAI14 ranked among the top 20% of transcripts in supporting‐cell populations, including Deiters and pillar cells, whereas MAP2, HAPLN1, and ANK1 ranked within the top 20% in spiral ganglion neuron populations (Jean et al. 2023; Supporting Information S3: Table 3). This distribution places several Group 3 components within cochlear compartments involved in epithelial support, structural organization, and auditory neural function, providing a cellular framework for interpreting the proteomic changes in relation to cytoskeletal, extracellular‐matrix, synaptic, and trafficking processes.

MAP2, PPP1R2, and SIRT2 were retained as dual‐assigned members of Groups 3 and 4 because their functions extend across structural organization and polarity‐associated regulation (Supporting Information S2: Table 2I). Their structural roles are emphasized here, whereas their regulatory functions are considered in Group 4. This functional overlap reflects the interconnected nature of the modules rather than independent effects of the same proteins.

From a translational perspective, MAP2, SIRT2, and SYNJ2 were classified as Tchem targets, whereas TUBB6 was classified as Tclin (Supporting Information S2: Table 2G). These annotations indicate pharmacological tractability but do not establish therapeutic efficacy in NIHL. SIRT2 inhibition has been reported to attenuate NIHL and reduce oxidative DNA damage and cochlear cell apoptosis in an experimental NIHL model (Liu et al. 2019), providing functional evidence that SIRT2 modulation can influence cochlear responses to acoustic injury. The present proteomic finding, however, shows reduced SIRT2 abundance, in contrast to the increased cochlear SIRT2 reported after noise exposure in that model, and therefore does not establish whether further modulation of SIRT2 would be beneficial or detrimental in the noise‐exposed cochlea, particularly given its context‐ and cell‐type‐dependent functions.

Overall, Group 3 defines a distributed structural interface linking cytoskeletal organization, extracellular matrix architecture, synaptic maintenance, and intracellular trafficking to the broader molecular response to acoustic stress. The reproducibility of eight proteins across an independent cochlear proteomic dataset, together with independent auditory and cellular evidence for selected components, strengthens the biological interpretation of this signature. The evidence supporting individual proteins was heterogeneous: HAPLN1 was supported by cochlear extracellular‐matrix localization and noise‐associated regulation; SYNJ2 by experimental auditory phenotypes and human HL associations; MYO1C by mechanotransduction biology; MAP2 by cochlear expression and an experimental hearing phenotype; and PRPH by experimental susceptibility to noise‐induced injury. Together, these findings support structural and synaptic remodeling as a component of the NIHL‐associated proteomic response and place the Group 3 signature within a broader network of cochlear and neuronal responses to acoustic stress.

### 4.6 Polarity Signaling and Cytoskeletal Dynamics Support Cochlear Architecture

Group 4 comprises six DAPs associated with regulatory processes related to cellular polarity, cytoskeletal organization, and signal‐dependent remodeling of cochlear cells. In contrast to Group 3, which is dominated by structural and synaptic components, Group 4 captures proteins whose functions are involved in the maintenance and remodeling of cellular architecture. The group includes the MAPK pathway kinase MAP2K2 (MEK2), the actin‐associated adaptor DBNL, the membrane‐associated regulator BASP1, and MAP2, PPP1R2, and SIRT2, which were also assigned to Group 3 because of their additional structural associations (Figure 3A; Supporting Information S2: Table 2C,I).

This regulatory signature was characterized by increased abundance of MAP2K2 and PPP1R2 and decreased abundance of BASP1, DBNL, MAP2, and SIRT2 (Supporting Information S2: Table 2A). The opposing directions of these changes are compatible with remodeling of polarity‐associated and cytoskeletal regulatory processes. The functional profile of the group included proteins associated with polarized cellular organization and cytoskeletal structures, while MAP2K2 contributes a distinct signaling component through the MAPK/ERK pathway.

BASP1 provides a relevant connection between polarity‐associated regulation and actin‐dependent cellular remodeling. BASP1 is a membrane‐associated protein with roles in actin organization, axonal growth, and synaptic plasticity (Chung et al. 2020). Its identification within regulatory networks associated with supporting‐cell‐to‐hair‐cell differentiation provides an additional context for its potential involvement in cellular plasticity (Kalra et al. 2023). Its downregulation in the present NIHL proteome is notable because BASP1 showed concordant downregulation in the independent cochlear proteomic dataset of Jongkamonwiwat et al. (2020) under one of the noise‐exposure conditions (Supporting Information S2: Table 2B). This cross‐study consistency supports the reproducibility of the BASP1 decrease across cochlear proteomic data sets. Its potential involvement in actin remodeling and cellular plasticity may be relevant to structural changes in the sensory epithelium following acoustic stress.

DBNL provides a complementary link between actin organization, protein trafficking, and epithelial polarity. DBNL is an activity‐associated adaptor that interacts with β‐catenin and contributes to N‐cadherin processing and adherens‐junction maturation, thereby supporting apico‐basal polarity (Herrera et al. 2021). These functions are relevant to the cochlear sensory epithelium, where adherens junctions contribute to the organization and integrity of the organ of Corti and its specialized cellular architecture. DBNL was downregulated in the present NIHL proteome. Its reduction at the protein level is consistent with changes in polarity‐associated structural regulation following acoustic stress.

The genetic context of DBNL should be interpreted separately from its functional annotation. *DBNL* was included among the human orthologs positioned relative to unresolved hereditary hHL loci in the integrative analysis (Supporting Information S2: Table 2D), but it should not be described as the gene underlying DFNB44. In the present positional analysis, *NUDCD3*, rather than *DBNL*, is located within the mapped DFNB44 interval, whereas the other Group 3 positional candidates are either outside or positioned relative to their corresponding intervals. Thus, the positional information for *DBNL* provides genetic context but does not support a specific DFNB44 association.

MAP2, discussed in Group 3 in relation to cochlear cytoskeletal organization, also contributes to Group 4 through its role in microtubule organization and cellular architecture. Its assignment to both groups reflects the multifunctional nature of MAP2, linking cytoskeletal structure with the spatial organization of cellular components. In this context, its reduced abundance in the present NIHL proteome may also reflect changes in the regulation of cellular architecture following acoustic stress.

SIRT2 provides an additional link between cytoskeletal regulation and cellular stress responses through its NAD+‐dependent deacetylase activity, including the regulation of α‐tubulin acetylation and microtubule dynamics. Through these functions, SIRT2 can influence cytoskeletal organization and other cellular processes associated with stress responses, although its specific contribution to cochlear structural regulation remains incompletely defined. Its reduced abundance in the present NIHL proteome therefore places SIRT2 within the cytoskeletal and regulatory component of the Group 4 signature.

PPP1R2 provides a regulatory link between protein phosphatase activity and cellular architecture. PPP1R2, also known as inhibitor‐2, is a regulatory interactor of protein phosphatase 1 (PP1), but its cellular function is more complex than its traditional designation as a PP1 inhibitor. Recent evidence indicates that PPP1R2 can stabilize specific PP1 holoenzymes and promote dephosphorylation of their substrates, thereby modulating phosphorylation‐dependent signaling (Lemaire et al. 2024). In addition, PPP1R2 has been implicated in the regulation of AURKA and PP1 at the centrosome, with experimental evidence linking its activity to central spindle organization and cytokinesis (Bresch et al. 2020). These findings provide a mechanistic basis for connecting PPP1R2 to cytoskeletal organization and cellular architecture. Its increased abundance in the present NIHL proteome is therefore compatible with altered regulation of phosphorylation‐dependent processes involved in cellular remodeling. However, increased PPP1R2 abundance does not by itself establish altered PP1 activity or changes in phosphorylation of its downstream substrates, which were not directly measured in the present proteomic analysis. Its assignment to both Groups 3 and 4 reflects its potential contribution to both structural and regulatory aspects of cellular organization.

MAP2K2 provides a direct link between Group 4 and MAPK/ERK signaling. MAP2K2 encodes MEK2, a central component of the RAF–MEK–ERK cascade. Pathogenic MAP2K2 variants cause cardiofaciocutaneous syndrome, a developmental disorder in which external‐ear abnormalities, hyperacusis, and HL have been reported (van Trier et al. 2015; Table 1 and Supporting Information S2: Table 2F). These clinical associations place MAP2K2 within developmental signaling pathways relevant to auditory and ear‐related phenotypes and provide additional biological context for the increased MAP2K2 abundance identified in the present NIHL proteome.

Recent experimental work provides additional context for considering MAP2K2 within the cochlear response to acoustic injury. KSR1, a scaffold that facilitates signaling through the BRAF–MEK1/2–ERK1/2 cascade, is expressed in the cochlea, and genetic disruption of KSR1 reduces MAPK phosphorylation and protects mice against noise‐induced hearing loss (Ingersoll et al. 2024). Pharmacological inhibition of the pathway likewise reduced noise‐ and cisplatin‐induced HL in that study. These findings establish a functional relationship between MAPK signaling and cochlear responses to acoustic injury and provide a biological context in which the increased MAP2K2 abundance observed here may be interpreted. They do not, however, demonstrate that MAP2K2 itself is activated in the present samples, because MEK2 phosphorylation, ERK activity, and downstream signaling were not measured.

The MAPK pathway may also intersect with cochlear cellular plasticity. MEK/ERK signaling is activated in supporting cells following hair‐cell injury, and experimental modulation of this pathway can alter supporting‐cell fate and hair‐cell differentiation in neonatal cochlear models (Ma et al. 2024). These findings indicate that MEK/ERK signaling can participate in both injury responses and cellular‐state transitions in the cochlear epithelium. In the present study, however, the increased abundance of MAP2K2 does not establish activation of this pathway or indicate a regenerative response, but places MAP2K2 within a signaling environment potentially relevant to cellular remodeling after acoustic stress.

The cellular distribution of Group 4 genes in the cochlear single‑cell atlas further links this regulatory signature to specific cochlear cell populations. Among the six genes, BASP1, MAP2, SIRT2, and PPP1R2 were highly ranked in supporting‑cell populations, particularly Deiters and pillar cells, and BASP1, MAP2, and SIRT2 also showed notable expression in glial populations (Jean et al. 2023; Supporting Information S2: Table 2). Supporting cells play essential roles in maintaining the organization, polarity, and structural integrity of the cochlear sensory epithelium and can exhibit cellular plasticity in response to injury, particularly during early postnatal development (Monzack and Cunningham 2013). This context is relevant to the present study because the animals were analyzed at P28–P30, a relatively early postnatal age in the mouse, although the cochlea is already functionally mature at this stage. The representation of multiple Group 4 genes in supporting‐cell populations therefore provides cellular context for a regulatory signature associated with cochlear architecture and plasticity. The atlas represents the basal transcriptomic state of the cochlea and therefore does not identify the cellular source of the protein changes observed after noise exposure.

From a translational perspective, MAP2K2 and TUBB6 were classified as Tclin proteins, whereas MAP2 and SIRT2 were classified as Tchem (Supporting Information S2: Table 2G). In the present, MAP2K2 and SIRT2 were increased and decreased in abundance, respectively, after noise exposure. MAP2K2 is associated with clinically used MEK inhibitors, indicating that the MEK/MAPK pathway contains pharmacologically tractable components. In a mouse model of NIHL, Ingersoll et al. (2024) showed that disruption of Ksr1, a scaffold regulating the BRAF–MEK1/2–ERK1/2 cascade, reduced MAPK phosphorylation and protected against NIHL, supporting the involvement of this pathway in acoustic injury. These findings provide translational context for the increased MAP2K2 protein abundance observed in the present study, although the Ingersoll et al. study did not specifically establish MAP2K2 as the therapeutic target. SIRT2 provides an additional translationally relevant example. In a mouse model of NIHL, Liu et al. (2019) reported increased SIRT2 protein abundance following noise exposure and showed that pharmacological inhibition of SIRT2 with AK‐7 attenuated NIHL, reduced oxidative DNA damage, and decreased cochlear cell apoptosis. In contrast, SIRT2 protein abundance was decreased in the present proteomic data set. This difference highlights the context‐dependent regulation of SIRT2 in acoustic injury and warrants further investigation. Pharmacological tractability should not be equated with therapeutic suitability for NIHL.

Overall, Group 4 represents a regulatory component of the NIHL‐associated proteomic response, encompassing proteins related to cellular polarity, cytoskeletal dynamics, phosphorylation‐dependent regulation, and stress‐responsive signaling. The mixed regulation of BASP1, DBNL, MAP2, SIRT2, PPP1R2, and MAP2K2, together with independent proteomic concordance for selected proteins and experimental evidence linking MAPK signaling to acoustic injury, supports the interpretation of Group 4 as a regulatory interface rather than a discrete pathway. The concordance of BASP1 and MAP2 with an independent cochlear proteomic data set and the cellular distribution of several Group 4 genes in cochlear supporting and glial populations provide additional context for this interpretation. Together, these findings suggest that acoustic stress is accompanied not only by changes in structural proteins, as described in Group 3, but also by changes in regulatory components associated with the maintenance and remodeling of cellular architecture and plasticity.

### 4.7 Specialized Regulatory and Immune‐Associated Proteins Expand the NIHL Molecular Landscape

Group 5 comprises 25 DAPs representing a heterogeneous set of regulatory, stress‐responsive, membrane‐associated, lysosomal, immune‐related, and tissue‐ or context‐specific proteins (Figure 3A,D; Supporting Information S2: Table 2C,I). Unlike the preceding groups, these proteins do not converge on a single dominant biological process. Instead, their organization into partially overlapping interaction modules suggests that Group 5 captures several cellular responses that may occur in parallel following acoustic injury, including protease regulation, membrane and vesicular remodeling, lysosomal processes, immune‐associated responses, and neurotrophic signaling. This heterogeneous organization consists of the cellular complexity of the cochlea and with the multiple forms of molecular stress elicited by acoustic trauma. Fifteen proteins were increased, and 10 were decreased in NIHL (Supporting Information S2: Table 2A). The principal PPI network comprised a protease‐regulatory/lysosomal module formed by A2M, SERPINA1B, SERPINA1C, SERPINA1E, SERPINA3K, and CTSD, together with a membrane‐associated module including ANXA5, ANXA6, ARFGAP2, and SEC. 22B (Figure 3D; Supporting Information S2: Table 2H). The remaining proteins showed more specialized or context‐dependent functions and were not incorporated into the principal network. Thus, Group 5 is better interpreted as a collection of interacting stress‐responsive processes rather than a single coordinated pathway.

The protease‐regulatory component is particularly notable because A2M and several SERPINA proteins were increased together with CTSD, a lysosomal aspartyl protease. The coexistence of multiple protease inhibitors with CTSD suggests remodeling of proteolytic homeostasis across extracellular and lysosomal compartments rather than a uniform change in proteolytic activity. The interaction between CTSD and A2M further connects lysosomal proteolysis with extracellular protease regulation within the Group 5 network. Accordingly, the coordinated abundance changes may reflect alterations in protein turnover and stress‐associated proteolytic balance following acoustic injury. Together, these changes indicate that protease regulation and lysosomal proteostasis are prominent components of the heterogeneous molecular response represented by Group 5.

CTSD provides additional auditory context for this lysosomal component. In a mouse model of chronic kanamycin ototoxicity, CTSD synthesis and activation increased during progressive cochlear hair‐cell loss, together with other nonclassical cell‐death responses (Jiang et al. 2006). Although aminoglycoside ototoxicity differs mechanistically from acoustic trauma, these findings provide direct evidence that CTSD can be engaged in cochlear hair‐cell injury and support the interpretation of its increased abundance here as part of a stress‐associated lysosomal response. Importantly, the present data do not establish that CTSD activation occurs in NIHL or that CTSD mediates hair‐cell loss.

The membrane‐associated component provides a complementary perspective. ANXA5 and ANXA6 were both reduced in the present NIHL proteome, together with ARFGAP2 and SEC. 22B, proteins involved in membrane trafficking and vesicle dynamics. ANXA5 is highly abundant in HCs stereocilia and has been proposed to participate in calcium‐dependent membrane repair (Bouter et al. 2011). However, genetic deletion of Anxa5 did not alter normal auditory function or the temporary or permanent hearing changes induced by noise exposure in mice (Krey et al. 2016). Thus, the reduction of ANXA5 observed here is more appropriately interpreted as evidence of membrane‐associated molecular remodeling than as evidence that impaired ANXA5‐dependent membrane repair contributes to NIHL. This finding illustrates that changes in protein abundance may reflect molecular remodeling without necessarily indicating that the affected protein is functionally required for the corresponding phenotype. ANXA6 provides a related link to membrane organization and intracellular signaling, although evidence from other forms of cochlear stress should be considered contextual rather than direct evidence of a role in NIHL. The presence of ANXA6 in extracellular vesicles released by cochlear hair cells following cisplatin exposure further provides evidence that ANXA6 is associated with membrane and extracellular‐vesicle responses during cochlear stress (Ai et al. 2025).

The independent proteomic dataset provides important support for selected components of this heterogeneous group. Fourteen Group 5 proteins were also detected as differentially regulated in the data set of Jongkamonwiwat et al. (2020) (Supporting Information S2: Table 2B). ANXA5 and ANXA6 showed concordant downregulation, whereas HRG IAH1 IGF2 and PRRC1 were concordantly increased. Other overlapping proteins showed divergent regulation, emphasizing that individual components of the stress response may vary according to exposure paradigm and temporal context. The concordant changes in annexins, HRG, IAH1, IGF2, and PRRC1 nevertheless provide independent support for selected Group 5 alterations and strengthen the reproducibility of these protein‐level changes across cochlear proteomic data sets.

PSAP provides one of the strongest auditory links within Group 5. Prosaposin is expressed throughout the organ of Corti and becomes localized to inner hair cells, supporting cells (Pillar and Deiters' cells), and the synaptic region of outer hair cells during postnatal development. In mice, prosaposin deficiency causes progressive HL, reduced distortion‐product otoacoustic emissions, outer hair‐cell abnormalities, and altered afferent and efferent cochlear innervation, supporting a role for PSAP in maintaining cochlear organization and auditory function (Akil et al. 2006). Consistent with this role, a prosaposin‐derived peptide has been shown to attenuate HL and inner hair‐cell damage in a model of transient cochlear ischemia (Terashita et al. 2013). The increased PSAP abundance observed (Supporting Information S2: Table 2A) here therefore occurs in a protein with established roles in cochlear function and neurotrophic signaling, providing a biologically relevant link between lysosomal/neurotrophic processes and cochlear stress responses. Together, these findings position PSAP as an informative component of the Group 5 signature linking lysosomal and neurotrophic biology to auditory function.

IGF2 provides another connection between Group 5 and developmental and cell‐survival processes. The increased abundance of IGF2 in the present proteome is notable because was also concordantly increased in the independent cochlear proteomic dataset, providing cross‐study support for this alteration (Supporting Information S2: Table 2B). Human alterations involving the 11p15.5 imprinting region that encompasses *IGF2* are associated with Beckwith–Wiedemann (MONDO#0007534, OMIM#130650) and Silver–Russell (MONDO#0014663, OMIM#616489) syndromes (Table 1 and Supporting Information S2: Table 2F), which can include external‐ear abnormalities (Zheng et al. 1997; Alberini 2023). Recent clinical evidence further indicates that hearing impairment can occur in Beckwith–Wiedemann syndrome, although reported abnormalities are predominantly related to middle‐ear structures and therefore do not establish a cochlear role for IGF2 itself (Parretta et al. 2026). Together, the concordant increase of IGF2 across cochlear proteomic data sets and its association with developmental disorders involving the 11p15.5 imprinting region provide developmental and auditory context for this Group 5 alteration.

The immune‐associated proteins IFI30, IGHM, HRG, and PSAP further indicate that Group 5 extends beyond intrinsic cochlear structural responses. In the Jean et al. (2023) atlas, *Ighm* was highly represented in B‐cell populations, whereas *Psap* and *Ifi30* showed representation in macrophage and monocyte populations. Other Group 5‐related genes were represented across additional nonsensory and sensory compartments, including *A2m* and *Anxa5* in lateral‐wall populations, *Anxa5* and *Clic6* in supporting‐cell populations, and *Anxa5* and *Gpx2* among more prominent transcripts in hair‐cell populations (Supporting Information S3: Table 3). This cellular distribution provides context for the heterogeneous protein composition captured by whole‐cochlear proteomics. The presence of immune‐associated transcripts in macrophage, monocyte, and B‐cell populations is also consistent with recent evidence that supporting cells can participate actively in inflammatory signaling following acoustic injury, including EGFR–ERK signaling and GSDMD‐dependent inflammatory responses associated with hair‐cell degeneration (Xiao et al. 2025). Conversely, *Ces1c*, *Hrg*, *Serpina1b, Serpina1c*, *Serpina1e*, and *Serpina3k* were not detected in the P20 atlas, precluding assignment of these proteins to specific cochlear cell populations based on this data set. Together, these observations place Group 5 proteins across immune, supporting, lateral‐wall, and sensory compartments, consistent with the heterogeneous cellular processes represented in this proteomic signature.

The genetic annotation of Group 5 also identifies proteins that may be relevant for prioritization within unresolved human hearing‐loss loci. *IAH1* and *PRRC1* correspond to human orthologs positioned within or relative to unresolved nonsyndromic hearing‐loss loci DFNB47 and DFNB60, respectively (Supporting Information S2: Table 2D). Their increased abundance in NIHL, together with concordant upregulation in the independent proteomic data set, provides convergent evidence supporting their prioritization for further genetic and functional investigation. These positional and proteomic associations place *IAH1* and *PRRC1* at the intersection of unresolved human HL genetics and an experimentally observed cochlear response to acoustic stress.

The neurotrophic and lysosomal components of Group 5 also intersect with broader mechanisms of neuronal maintenance. The interaction between PSAP and CTSD places two proteins involved in lysosomal and neurotrophic biology within the same PPI network, while PSAP has established effects on cochlear innervation and auditory function (Akil et al. 2006). This network connection provides an additional link between lysosomal proteostasis and the maintenance of cochlear neural and epithelial compartments during acoustic stress.

From a translational perspective, Group 5 contains proteins with different levels of pharmacological tractability. CES1C and CTSD were classified as Tchem proteins, whereas EPPK1, PRRC1, and SERPINA3K were classified as Tdark in the present annotation framework (Supporting Information S2: Table 2G). These classifications indicate differing levels of available chemical or biological information. The Tchem designation of CTSD and CES1C identifies proteins with documented chemical tractability, whereas the Tdark classification of PRRC1 and SERPINA3K reflects comparatively limited functional characterization, making these proteins less suitable for immediate pharmacological prioritization.

From a translational perspective, Group 5 contains proteins with different levels of pharmacological tractability. CES1C and CTSD were classified as Tchem proteins, whereas EPPK1, PRRC1, and SERPINA3K were classified as Tdark in the present annotation framework (Supplementary Table 2G). These classifications indicate differing levels of available chemical or biological information. The Tchem designation of CTSD and CES1C identifies proteins with documented chemical tractability, whereas the Tdark classification of PRRC1 and SERPINA3K reflects comparatively limited functional characterization. Together, these annotations provide a translational framework for prioritizing proteins within the heterogeneous Group 5 signature for future mechanistic and pharmacological investigation.

### 4.8 Study Limitations

Several limitations should be considered when interpreting the present findings. First, the proteomic analysis was performed using whole‐cochlear homogenates, and each biological replicate consisted of pooled cochleae from two animals. Consequently, the measured protein abundances represent the integrated contribution of multiple cochlear cell populations and do not allow direct resolution of cell‐type‐specific or spatial protein changes. Integration with publicly available single‐cell transcriptomic data provided additional cellular context, but these data do not establish the cellular origin of the corresponding protein‐level changes. Cell‐type‐resolved and spatial proteomic approaches will therefore be important to determine how the identified molecular alterations are distributed across cochlear compartments.

Second, the study did not include experimental validation of selected proteins by immunoblotting, immunohistochemistry, targeted proteomics, or functional assays. The identified protein changes therefore provide a molecular framework for further investigation, while their effects on protein function, pathway activity, and cochlear injury remain to be determined. Targeted protein validation and functional studies, including assessment of protein localization and activity where appropriate, will be important for establishing the biological significance of the prioritized candidates.

Third, the integration of mouse proteomic data with human deafness loci provides a framework for biological and positional prioritization. The correspondence between a differentially abundant mouse protein and a human deafness locus is informative for candidate prioritization, particularly when the underlying locus contains multiple plausible genes, but does not identify the gene responsible for the human phenotype. These associations therefore provide candidate biological links for further investigation of the relationship between molecular changes and auditory phenotypes.

Finally, functional enrichment and network analyses provide complementary biological context for the observed protein changes. Their interpretation is based on the convergence of protein abundance patterns with established biological functions and independent transcriptomic and genetic evidence, while PPI networks reflect reported or predicted molecular associations. Future studies combining cell‐type‐resolved proteomics, targeted validation, and functional approaches will be important to define the cellular specificity, temporal behavior, and mechanistic significance of the identified molecular alterations.

Despite these limitations, the integration of quantitative proteomics with transcriptomic and human genetic information provides a framework for prioritizing proteins and biological processes associated with noise‐induced cochlear injury and for guiding future mechanistic studies.

## 5 Conclusions and Future Directions

This study provides a systems‐level proteomic characterization of the adult mouse cochlea following noise exposure, identifying a NIHL‐associated signature comprising 49 quantitatively defined DAPs and 31 condition‐specific proteins. These proteins were organized into five functional groups encompassing metabolic and redox processes, RNA‐related regulation, structural and synaptic organization, polarity and cytoskeletal signaling, and specialized regulatory, immune‐associated, lysosomal, and membrane‐related functions. Together, these findings indicate that acoustic trauma is accompanied by coordinated changes across multiple molecular processes in the cochlea.

Integration with functional enrichment, PPI analysis, an independent cochlear proteomic data set, cochlear single‐cell transcriptomic context, and human genetic annotation provided complementary support for the biological interpretation of the proteomic signature and enabled prioritization of selected proteins for further investigation. The cross‐study comparison further highlighted both reproducible and context‐dependent protein responses, while transcriptomic and genetic information provided additional cellular and biological context for selected components of the signature.

Future studies combining cell‐type‐resolved or spatial proteomics, targeted protein validation, longitudinal analyses, and functional approaches will be important to define the cellular specificity, temporal behavior, and mechanistic significance of the identified molecular changes. Such studies may help determine how these alterations contribute to cochlear vulnerability and recovery following acoustic trauma and advance the present proteomic framework toward a more mechanistic understanding of NIHL.

## Author Contributions

Design research: All authors contributed to the study conception and design. Experimental data: Ana Carla Batissoco and Karina Lezirovitz. Computational analyses: Ana Carla Batissoco, Danillo Alencar‐Coutinho, Ana Carolina Ferreira‐Scatone, Stella Diogo Cavassana, and Karina Lezirovitz. Funding acquisition: Ana Carla Batissoco, Jeanne Oiticica, Karina Lezirovitz, and Ricardo Ferreira Bento. Project administration: Ana Carla Batissoco, Jeanne Oiticica, Karina Lezirovitz, and Ricardo Ferreira Bento. Resources: Ana Carla Batissoco, Jeanne Oiticica, Karina Lezirovitz, and Ricardo Ferreira Bento. Manuscript writing: Ana Carla Batissoco. Review and editing: Ana Carla Batissoco, Karina Lezirovitz, and Ricardo Ferreira Bento. All authors read and approved the final manuscript.

## Conflicts of Interest

The authors declare that no conflicts of interest.

## Ethics Statement

All experimental procedures were approved by the Institutional Animal Care and Use Committee of the University of São Paulo School of Medicine (protocol numbers 021/2017, 022/2017, and 1764/2022) and were conducted in accordance with the guidelines for the care and use of laboratory animals established by the National Research Council (USA).

## Supporting information


**Table S1:** Global cochlear proteome profiling in normal hearing (NH) and noise‐induced hearing loss (NIHL) mice.

**Table S2:** Functional, genetic, and pharmacological annotation of differentially abundant proteins associated with noise‐induced hearing loss (NIHL).

**Table S3:** Single‐cell transcriptomic analysis of NIHL‐associated differentially abundant protein‐encoding genes across cochlear cell types.

## Data Availability

The mass spectrometry–based proteomics data generated in this study have been deposited in the ProteomeXchange Consortium via the PRIDE repository under the accession number PXD058223. All other data supporting the findings of this study are included in the manuscript and its Supporting Information.
